# MdNAC104 positively regulates apple cold tolerance via CBF‐dependent and CBF‐independent pathways

**DOI:** 10.1111/pbi.14112

**Published:** 2023-06-30

**Authors:** Chuang Mei, Jie Yang, Quanlin Mei, Dongfeng Jia, Peng Yan, Beibei Feng, Aisajan Mamat, Xiaoqing Gong, Qingmei Guan, Ke Mao, Jixun Wang, Fengwang Ma

**Affiliations:** ^1^ State Key Laboratory of Crop Stress Biology for Arid Areas/Shaanxi Key Laboratory of Apple, College of Horticulture Northwest A & F University Yangling Shaanxi China; ^2^ The State Key Laboratory of Genetic Improvement and Germplasm Innovation of Crop Resistance in Arid Desert Regions (Preparation), Key Laboratory of Genome Research and Genetic Improvement of Xinjiang Characteristic Fruits and Vegetables, Institute of Horticulture Crops Xinjiang Academy of Agricultural Sciences Urumqi China

**Keywords:** *Malus domestica*, Cold tolerance, MdNAC104, CBF pathway, Anthocyanin, ROS scavenging

## Abstract

Low temperature is the main environmental factor affecting the yield, quality and geographical distribution of crops, which significantly restricts development of the fruit industry. The NAC (NAM, ATAF1/2 and CUC2) transcription factor (TF) family is involved in regulating plant cold tolerance, but the mechanisms underlying these regulatory processes remain unclear. Here, the NAC TF MdNAC104 played a positive role in modulating apple cold tolerance. Under cold stress, *MdNAC104*‐overexpressing transgenic plants exhibited less ion leakage and lower ROS (reactive oxygen species) accumulation, but higher contents of osmoregulatory substances and activities of antioxidant enzymes. Transcriptional regulation analysis showed that MdNAC104 directly bound to the *MdCBF1* and *MdCBF3* promoters to promote expression. In addition, based on combined transcriptomic and metabolomic analyses, as well as promoter binding and transcriptional regulation analyses, we found that MdNAC104 stimulated the accumulation of anthocyanin under cold conditions by upregulating the expression of anthocyanin synthesis‐related genes, including *MdCHS‐b*, *MdCHI‐a*, *MdF3H‐a* and *MdANS‐b*, and increased the activities of the antioxidant enzymes by promoting the expression of the antioxidant enzyme‐encoding genes *MdFSD2* and *MdPRXR1.1*. In conclusion, this study revealed the MdNAC104 regulatory mechanism of cold tolerance in apple via CBF‐dependent and CBF‐independent pathways.

## Introduction

Temperature is one of the main environmental factors affecting plant growth and development. Cold stress, including chilling stress (0–15 °C) and freezing stress (<0 °C), adversely affect crop yield and quality and significantly restricts geographical distribution (Ding and Yang, 2022; Guo *et al*., 2018; Thomashow, 1999). Cell membrane structures are damaged by low temperatures, and the metabolism of nutrients is disrupted, leading to inhibited growth or death (Burke *et al*., 1976; Ding *et al*., 2019). Plants have evolved complex and elaborate regulatory mechanisms to rapidly sense and effectively respond to cold stress. One of the main mechanisms that plants respond and adapt to cold stress is the DREB1/CBF (DRE binding factor 1/CRT binding factor)‐dependent cold signalling pathway (Aslam *et al*., 2022; Thomashow, 1999). Once exposed to cold conditions, the expression of CBF transcription factor (TF) family genes, such as *CBF1* and *CBF3* in *Arabidopsis*, is rapidly activated, and CBF proteins accumulate. These CBF TFs activate the transcription of downstream *cold‐responsive* (*COR*) genes to transmit and amplify the cold signal; thus, triggering a series of physiological responses (Kidokoro *et al*., 2022; Liu *et al*., 2019). Studies in other plant species indicate that this CBF‐COR pathway is well‐conserved, such as *Arabidopsis* (Liu *et al*., 2019), rice (Li *et al*., 2022b; Nakamura *et al*., 2011), tomato (Wang *et al*., 2018a) and apple (An *et al*., 2018a; Feng *et al*., 2012; Xie *et al*., 2018; Yang *et al*., 2023). In apples, five DREB1/CBF family genes were identified (Feng *et al*., 2012). Cold stress significantly induces the expression of these *MdCBFs* and their target *COR* genes, such as *MdKIN1*, *MdRD29A* and *MdCOR47* (An *et al*., 2018a; Xie *et al*., 2018). Ectopic expression of *MbDREB1* from dwarf apple in *Arabidopsis* or *PbCBF1* from peach in apple significantly enhance the cold tolerance of transgenic plants (Wisniewski *et al*., 2015; Yang *et al*., 2011). In addition, jasmonic acid (JA), ethylene, abscisic acid (ABA) and calcium signalling‐mediated cold tolerance in apple is related to the upregulation of these *MdCBFs* and *COR* genes (An *et al*., 2020, 2022; Wang *et al*., 2021b; Yang *et al*., 2023).

Due to the important roles of *CBF* genes in the plant cold response, various TFs that directly regulate their expression have been identified, such as the well‐known positive regulator ICE1 (Inducer of CBF Expression 1) and the negative regulator MYB15 in *Arabidopsis* (Agarwal *et al*., 2006; Chinnusamy *et al*., 2003). In apples, several TFs that directly regulate the expression of *CBF* genes and thus cold tolerance have also been identified, including the bHLH TFs MdCIbHLH1/MdICE1, MdbHLH33, MdICE1L and MdbHLH4 (An *et al*., 2020, 2022; Feng *et al*., 2012; Xu *et al*., 2018a; Yang *et al*., 2023), the MYB TFs MdMYB15L and MdMYB23 (An *et al*., 2018b; Xu *et al*., 2018b), the ERF (ethylene responsive factor) TF MdERF1B (Wang *et al*., 2021b), the bZIP TF MdHY5 (An *et al*., 2017), the B‐box (BBX) TF MdBBX37 (An *et al*., 2020) and the NAC (NAM, ATAF1/2 and CUC2) TF MdNAC029/MdNAP (An *et al*., 2018a). The NAC family is one of the largest TF families in plants, and its members are characterized by the well‐conserved N‐terminal NAC domain (Olsen *et al*., 2005; Puranik *et al*., 2012). The NAC family plays diverse roles in regulating plant growth and development and the responses to abiotic stressors, including drought, salt and cold (An *et al*., 2018a; Qu *et al*., 2016; Souer *et al*., 1996; Yu *et al*., 2015). NAC TFs are involved in modulating the cold response by regulating cell membrane flow, synthesis of hormones and the antioxidant system (Hu *et al*., 2008; Souer *et al*., 2015). Studies on NAC TFs in other plant species suggest that some NAC proteins regulate the cold stress response through the CBF‐COR pathway, such as SlNAC35 in tomato (Wang *et al*., 2018a), PbeNAC1 in pear (Jin *et al*., 2017) and GmNAC20 in soybean (Hao *et al*., 2011). However, the mechanisms underlying NAC‐mediated cold signalling remain unclear. In apples, MdNAC029 negatively regulates cold tolerance by directly binding to the *MdCBF1* and *MdCBF4* promoters and inhibiting their expression (An *et al*., 2018a). Other NAC proteins that are involved in regulating *CBF* expression and cold tolerance in apples have not been explored.

Cold stress damages cell membrane structure, leading to decreased extracellular water potential, increased ion leakage and malondialdehyde (MDA) content, as well as overproduction of reactive oxygen species (ROS) (Ding *et al*., 2019; Qi *et al*., 2018). Plants have evolved various physiological mechanisms to alleviate damage caused by cold stress, which promote the accumulation of osmoregulatory substances and improve the activity of antioxidant enzymes (Hoermiller *et al*., 2022; Wang *et al*., 2020; Xu *et al*., 2022b). The accumulation of dehydrins and osmotic regulating substances, such as soluble sugars, soluble proteins, betaine and proline, helps to reduce osmotic pressure and stabilize cell membranes, thus improving plant tolerance to cold stress (Feng *et al*., 2013; Hayashi *et al*., 1997; Hoermiller *et al*., 2022; Meena *et al*., 2019; Xin and Browse, 2010). Stress‐induced ROS signalling plays an important role in the regulation of the stress response (Mittler *et al*., 2022; Wang *et al*., 2018c). However, excessive accumulation of ROS damages proteins, lipids and nucleic acids, leading to cell death in severe cases (Noctor *et al*., 2014). Plants have evolved enzymatic and non‐enzymatic scavenging systems to eliminate excessive ROS. Superoxide dismutase (SOD), peroxidase (POD) and catalase (CAT) are the primary enzymes within the enzymatic scavenging system, while non‐enzymatic antioxidants include ascorbic acid, carotenoids and flavonoids (Aslam *et al*., 2022; Mittler *et al*., 2022; Noctor *et al*., 2014; Wang *et al*., 2018c). Until now, many TFs have been shown to regulate stress tolerance in plants by affecting ROS scavenging, such as bHLHs (Luo *et al*., 2020), MYBs (Xie *et al*., 2018), DREBs (Liang *et al*., 2022) and NACs (Han *et al*., 2020). However, the mechanisms underlying the regulation of the antioxidant enzyme activities mediated by these TFs in response to cold are largely unknown.

Anthocyanins are secondary metabolites that improve cold stress tolerance by scavenging free radicals and reducing oxidative damage (Jin *et al*., 2022; Van Den Ende and El‐Esawe, 2014). Anthocyanins are biosynthesized through the flavonoid pathway, including the enzymes chalcone synthase (CHS), chalcone isomerase (CHI), flavanone 3‐hydroxylase (F3H), dihydroflavonol 4‐reductase (DFR) and anthocyanidin synthase (ANS) (Hichri *et al*., 2011; Koes *et al*., 2005). A variety of TFs affect anthocyanin accumulation and cold tolerance in apples. For example, MdMYB2 affects anthocyanin accumulation and cold tolerance by activating the SUMO E3 ligase MdSIZ1 (Jiang *et al*., 2022); MdbHLH33 positively regulates cold tolerance and anthocyanin accumulation in apple by activating the expression of MdCBF2 and MdDFR (Xu *et al*., 2017, 2018a); MdMYB88 and MdMYB124 confer enhanced cold tolerance by promoting hydrogen peroxide (H_2_O_2_) scavenging and the accumulation of anthocyanin (Xie *et al*., 2018). NAC TFs play an important role in the biosynthesis and accumulation of anthocyanins (Jiang *et al*., 2019; Zhou *et al*., 2015). For example, AtNAC078 promotes anthocyanin accumulation by stimulating the transcription of *AtPAP1*, *AtCHS*, *AtCHI* and *AtF3H* in response to strong light stress (Morishita *et al*., 2009). BoNAC019 reduces anthocyanin accumulation by inhibiting the expression of anthocyanin synthesis genes, leading to an increase in ROS and a decrease in drought resistance in *Brassica oleracea* (Wang *et al*., 2018b); LcNAC90 promotes the synthesis of anthocyanins by directly regulating the transcription of *LcPAL* and *LcCHS* in *Litchi chinensis* in response to ABA and GA3 during storage (Qu *et al*., 2022). In apples, MdNAC52 improves light‐induced anthocyanin accumulation by upregulating *MdMYB9* and *MdMYB11* expression (Sun *et al*., 2019), and MdNAC42 promote anthocyanin synthesis by interacting with MdMYB10 under UV‐B irradiation (Zhang *et al*., 2020). However, whether NAC TFs regulate cold tolerance by directly regulating anthocyanin biosynthesis in response to cold stress is unclear.

Cold stress is a primary environmental factor that constrains development of the fruit tree industry. Apples are a commercially important fruit tree, and cold stress significantly affects the yield and quality as well as limits the geographical distribution of apples. In our previous study, we identified the NAC transcription factor MdNAC104, which is involved in the regulation of apple dwarfing (Jia *et al*., 2018) and drought tolerance (Jia *et al*., 2019). However, its function in the cold stress response is unclear. In this study, a positive role of MdNAC104 in regulating cold stress tolerance was identified using *MdNAC104‐*overexpressing transgenic apple plants. Through transcriptome and metabolome analysis, as well as promoter binding and transcriptional regulation analyses, the regulatory mechanisms underlying MdNAC104‐mediated cold tolerance in apple plants were clarified by promoting *MdCBF* expression, anthocyanin accumulation, and antioxidant enzyme activities. This study provides insight into the role of NAC TFs in the regulation of the apple cold response through CBF‐dependent and CBF‐independent pathways.

## Results

### Overexpressing *MdNAC104* enhances cold tolerance in transgenic apple plants

In previous studies, we identified the NAC TF gene *MdNAC1* (MF401514.1 and MD15G1415700) that regulates apple dwarfing and drought tolerance (Jia *et al*., 2018, 2019). To distinguish this gene from the recently published *MdNAC1* (MD10G1198400) (Li *et al*., 2022a) (Figure S1), we renamed the gene that we cloned *MdNAC104* following its orthologues in other plants (Jia *et al*., 2018). To study the response of *MdNAC104* to cold stress, tissue‐cultured ‘GL‐3’ apple plants were treated at 4 °C for different time periods. The expression of *MdNAC104* was significantly induced by the cold treatment and rapidly reached a peak within 3 h (Figure S2a). To further verify the effect of cold stress on *MdNAC104* transcription, the *MdNAC104* promoter was cloned and used to drive expression of the *gusA* gene. The GUS staining and activity measurements showed that the cold stress significantly promoted *MdNAC104* promoter transcriptional activity (Figure S2b,c).

**Figure 1:**
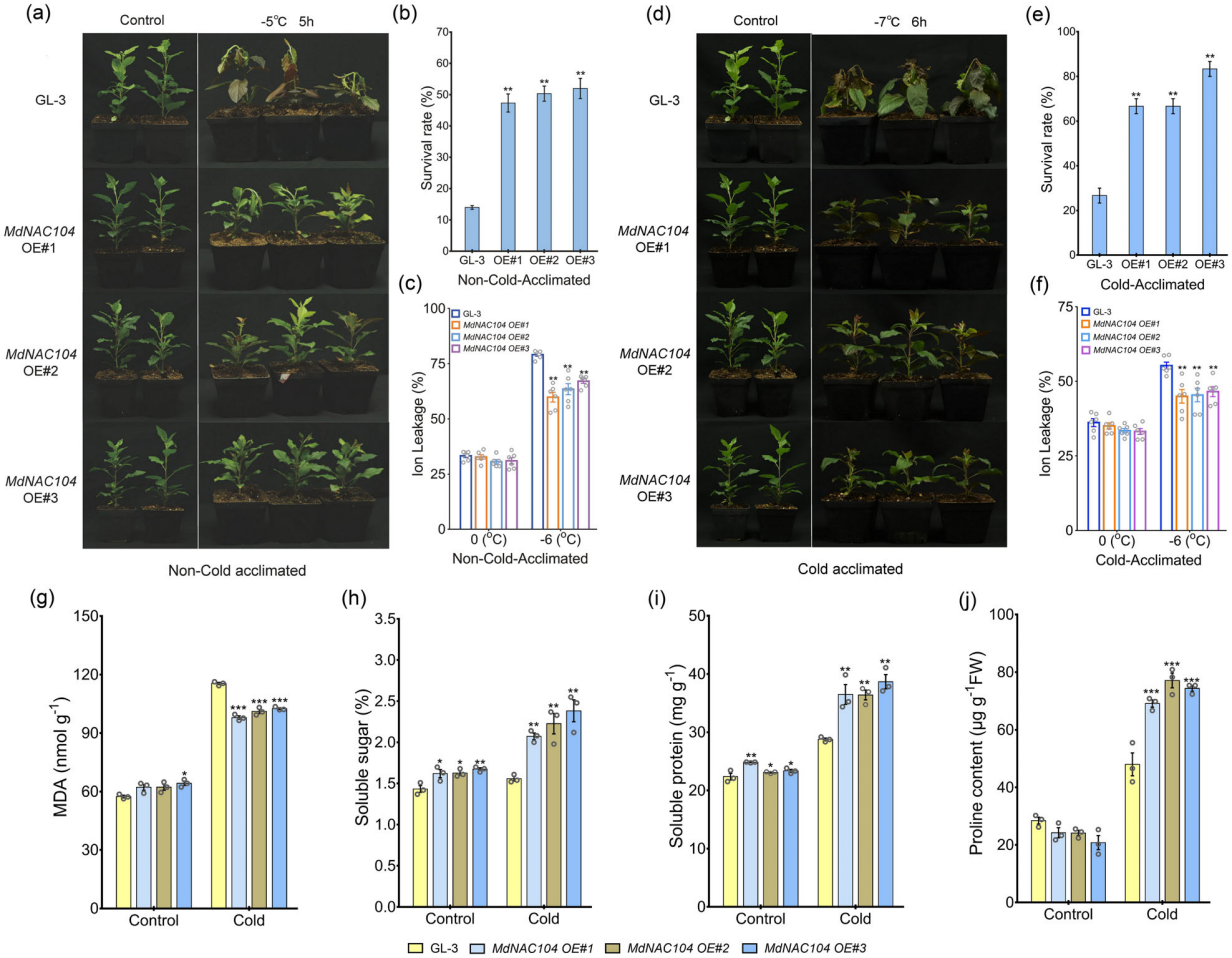
Overexpressing *MdNAC104* enhances cold tolerance in transgenic apple plants. (a) The phenotypes of the GL‐3 and *MdNAC10*4 transgenic plants in the NA group after the freezing treatment (−5 °C, 5 h). (b) Survival rates. (c) Relative electrolyte leakage (REL). (d) The phenotypes of the GL‐3 and *MdNAC10*4 transgenic plants in the CA group after the freezing treatment (−7 °C, 6 h). (e) Survival rates. (f) REL. (g) MDA content. (h) Soluble sugar content. (i) Soluble protein content. (j) Proline content. Error bars indicate the SE of three biological replicates. One‐way ANOVA (Tukey's test) was performed, and significant differences are indicated by: *P < 0.05; **P < 0.01; ***P < 0.001.

*MdNAC104*‐overexpressing transgenic plants were treated with a freezing treatment to identify the function of *MdNAC104* in modulating apple cold tolerance. The materials were divided into the non‐cold‐acclimated (NA) and cold‐acclimated (CA) groups. The transgenic and GL‐3 plants in the NA group were exposed to −5 °C for 5 h. After the treatment, the leaves of the GL‐3 plants had large brown spots, and most of the leaves had wilted or died, while the *MdNAC104* transgenic plants only wilted slightly and the plants had red spots on the top young leaves, indicating that the transgenic plants suffered less stress damage (Figure 1a). The survival rate and REL measurements supported this result, with a significantly higher survival rate and a lower REL detected in the transgenic lines than GL‐3 after the freezing treatment (Figure 1b,c). The transgenic and GL‐3 apple plants in the CA group were exposed for 72 h at 4 °C for the cold acclimation, then subjected to −7 °C for 6 h. After the treatment, the leaves of the upper half of the GL‐3 plants were wilted, with large brown spots on the leaves. In contrast, most of the leaves on the transgenic plants were vigorous (Figure 1d). The survival rate and REL of the transgenic lines were also, respectively, higher and lower than those of the GL‐3 plants after the freezing treatment (Figure 1e,f). These results indicate that overexpressing *MdNAC104* enhanced cold tolerance in transgenic apple plants.

To further analyse the effect of cold stress on the apple plants, the contents of MDA and osmotic adjusting substances, for example soluble sugars, soluble proteins and proline, were determined in the leaves of GL‐3 and transgenic plants (CA group). After the freezing treatment, the MDA contents in the leaves of the transgenic lines were significantly lower than those in the GL‐3 plants (Figure 1g), while soluble sugar, soluble protein and proline contents were significantly higher than those in GL‐3 plants (Figure 1h,j). Additionally, the levels of soluble sugars and soluble proteins in the transgenic plants were also slightly higher than those of the GL‐3 plants under normal conditions (Figure 1h,i). This result indicates that MdNAC104 may enhance the cold tolerance of apple plants by regulating the accumulation of osmotic adjusting substances.

### Overexpressing *MdNAC104* inhibits excess ROS accumulation by increasing antioxidant enzyme activities

**Figure 2:**
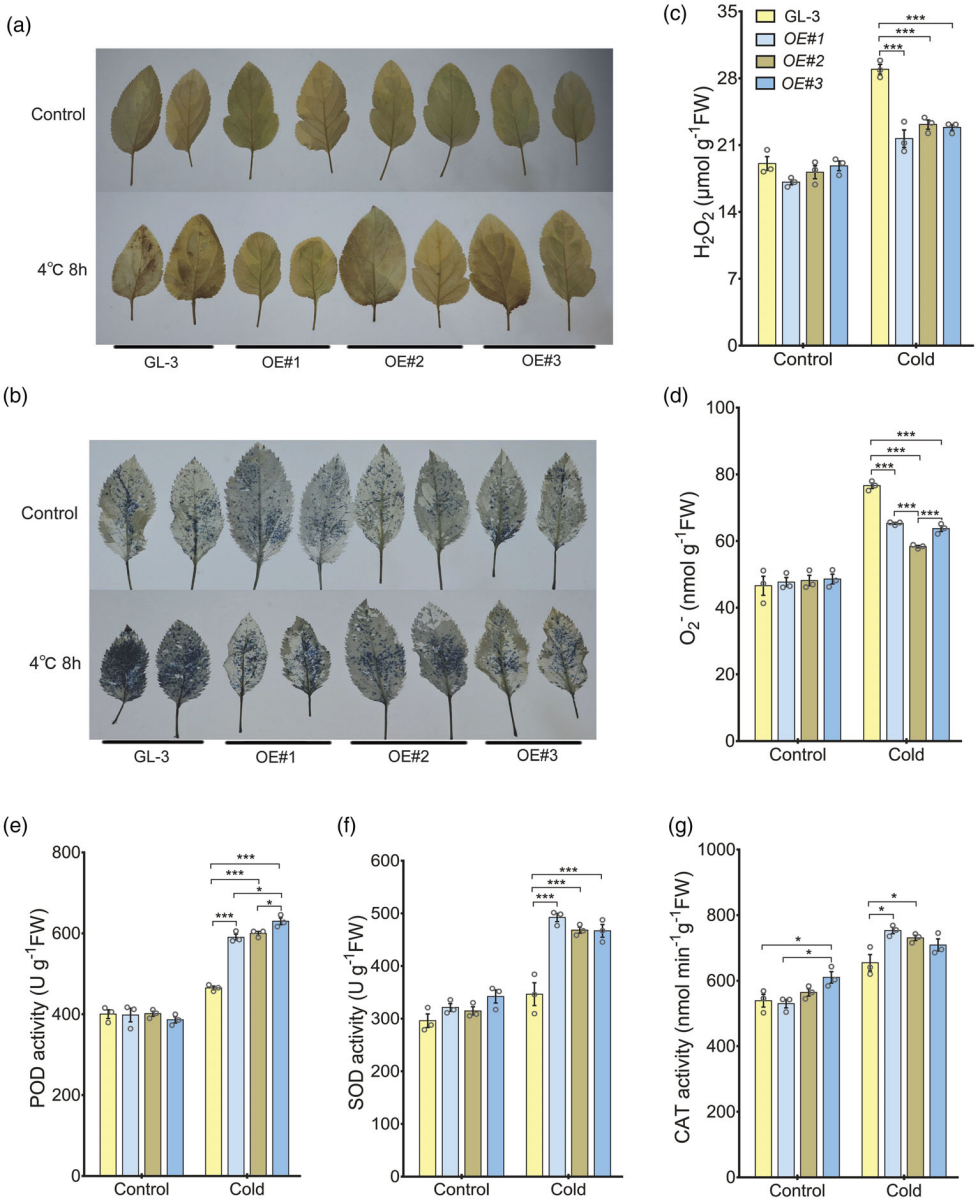
ROS accumulation and the antioxidant enzyme activities in leaves of GL‐3 and *MdNAC104* transgenic apple plants. (a) DAB staining of H_2_O_2_ in leaves. (b) NBT staining of O_2_^−^ in leaves. (c) H_2_O_2_ content. (d) O_2_^−^ content. (e) POD activity. (f) SOD activity. (g) CAT activity. Error bars indicate the SE of three biological replicates. One‐way ANOVA (Tukey's test) was performed, and significant differences are indicated by: *P < 0.05; ***P < 0.001.

The freezing treatment resulted in the wilting and death of most of the GL‐3 leaves, which was not conducive for a detailed analysis of the cold response in GL‐3 and *MdNAC104* transgenic plants. To compare the leaf indices of the different lines under cold stress, apple plants were treated at 4 °C for 8 h for subsequent histochemical staining and determination of physiological indices. DAB and NBT histochemical staining were performed to investigate ROS accumulation in the leaves of *MdNAC104* transgenic and GL‐3 plants. Staining of the leaves of the GL‐3 and transgenic lines was not significantly different under normal conditions (Figure 2a,b). After the cold treatment, the GL‐3 leaves had large brown and dark blue spots under DAB and NBT staining, respectively, whereas significantly fewer stained spots were observed on the leaves of the transgenic plants (Figure 2a,b), indicating lower accumulation of H_2_O_2_ and O^2−^ in the transgenic plants than the GL‐3 plants. The H_2_O_2_ and O^2−^ measurements further verified the histochemical staining results (Figure 2c,d). These results suggest that overexpressing *MdNAC104* significantly decreased the accumulations of ROS in apple leaves under cold stress.

Antioxidant enzymes, such as POD, SOD and CAT, promote the removal of ROS, thereby alleviating the damage caused by excess ROS accumulation under abiotic stress conditions (Dong *et al*., 2022). The antioxidant enzyme activities in the leaves of the *MdNAC104* transgenic and GL‐3 plants were investigated. As results, POD, SOD and CAT activities were similar among the lines under normal conditions. However, the activities of these enzymes in the *MdNAC104* transgenic lines were significantly higher than those in GL‐3 plants after the cold treatment, particularly POD and SOD (Figure 2e–g). These results indicate that overexpressing *MdNAC104* increased antioxidant enzyme activities in apples under cold stress, thereby inhibiting excess ROS accumulation.

### MdNAC104 promotes *MdCBF1* and *MdCBF3* expression under cold conditions by directly binding to their promoters

**Figure 3:**
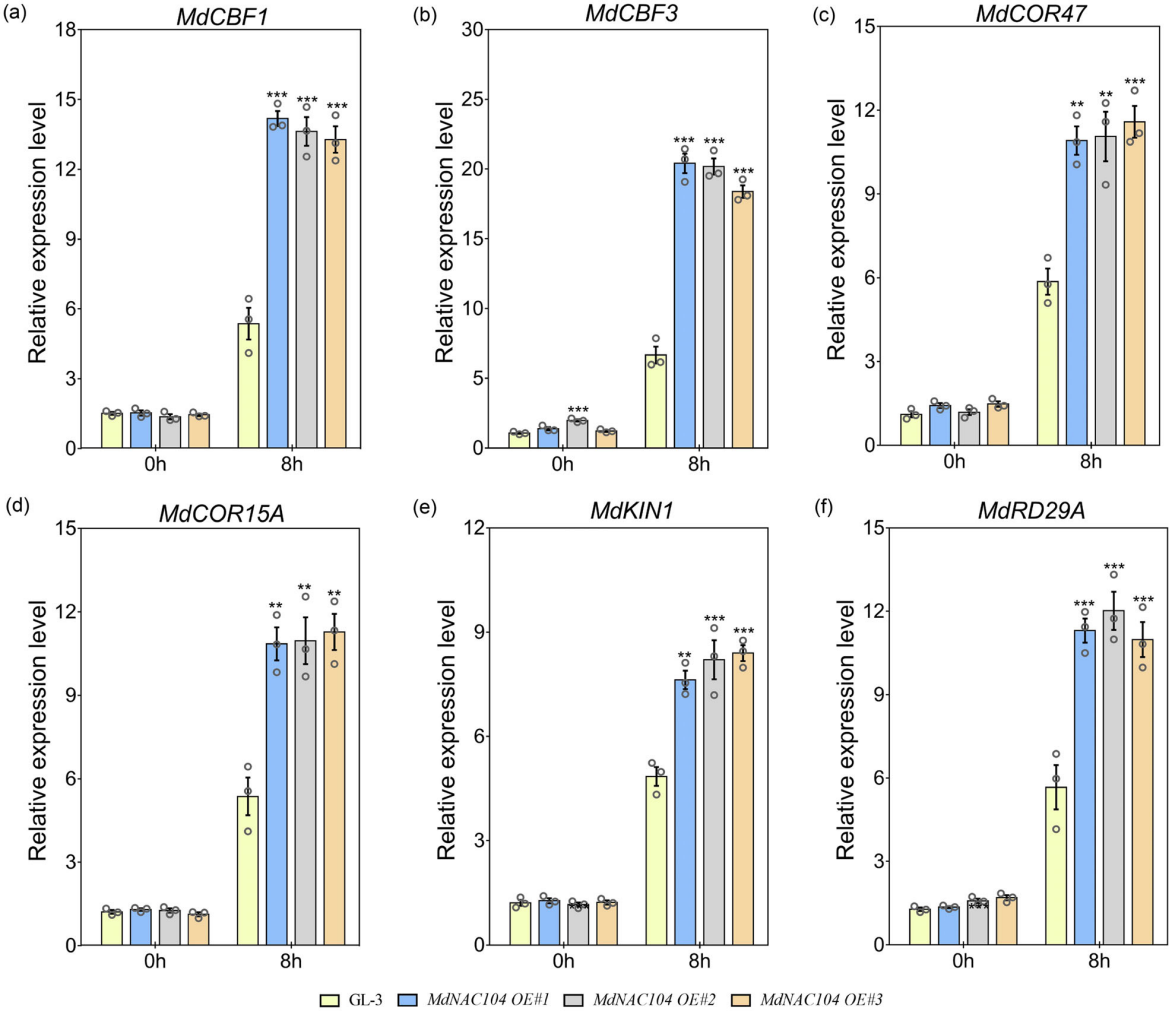
Expression analysis of the cold response genes in GL‐3 and *MdNAC104* transgenic apple plants under cold stress. The relative expression levels of *MdCBF1* (a), *MdCBF3* (b), *MdCOR47* (c), *MdCOR15A* (d), *MdKIN1* (e), and *MdRD29A* (f) were shown. Error bars indicate the SE of three biological replicates. One‐way ANOVA (Tukey's test) was performed, and significant differences are indicated by: *P < 0.05; **P < 0.01; ***P < 0.001.

The CBF‐COR pathway has been demonstrated to be a key mechanism in the cold response. This pathway responds to low temperature and regulates several physiological and biochemical processes related to the cold response, including antioxidant enzyme activity and the accumulation of osmoregulatory substances (Li *et al*., 2022b; Wang *et al*., 2021a). In *Arabidopsis*, the CBF‐COR pathway includes several cold‐responsive genes, such as *CBF1* and *CBF3*, as well as their downstream target genes *RD29A*, *COR15A*, *COR47* and *KIN1* (Aslam *et al*., 2022; Ding and Yang, 2022; Kidokoro *et al*., 2022). The orthologs of these genes in apples were also shown to significantly respond to cold stress (An *et al*., 2018a; Feng *et al*., 2012; Wang *et al*., 2021b; Xie *et al*., 2018; Yang *et al*., 2023). The expression changes in these cold‐responsive genes were analysed in *MdNAC104* transgenic and GL‐3 plants to clarify the MdNAC104 mechanism of the apple cold stress response. The cold treatment significantly increased the expression levels of these genes in the transgenic and GL‐3 plants. Furthermore, the expression levels of these genes were significantly higher in the *MdNAC104* transgenic lines than in GL‐3 under cold stress (Figure 3a–f), indicating that MdNAC104 positively regulates the expression of cold response genes under cold conditions.

**Figure 4:**
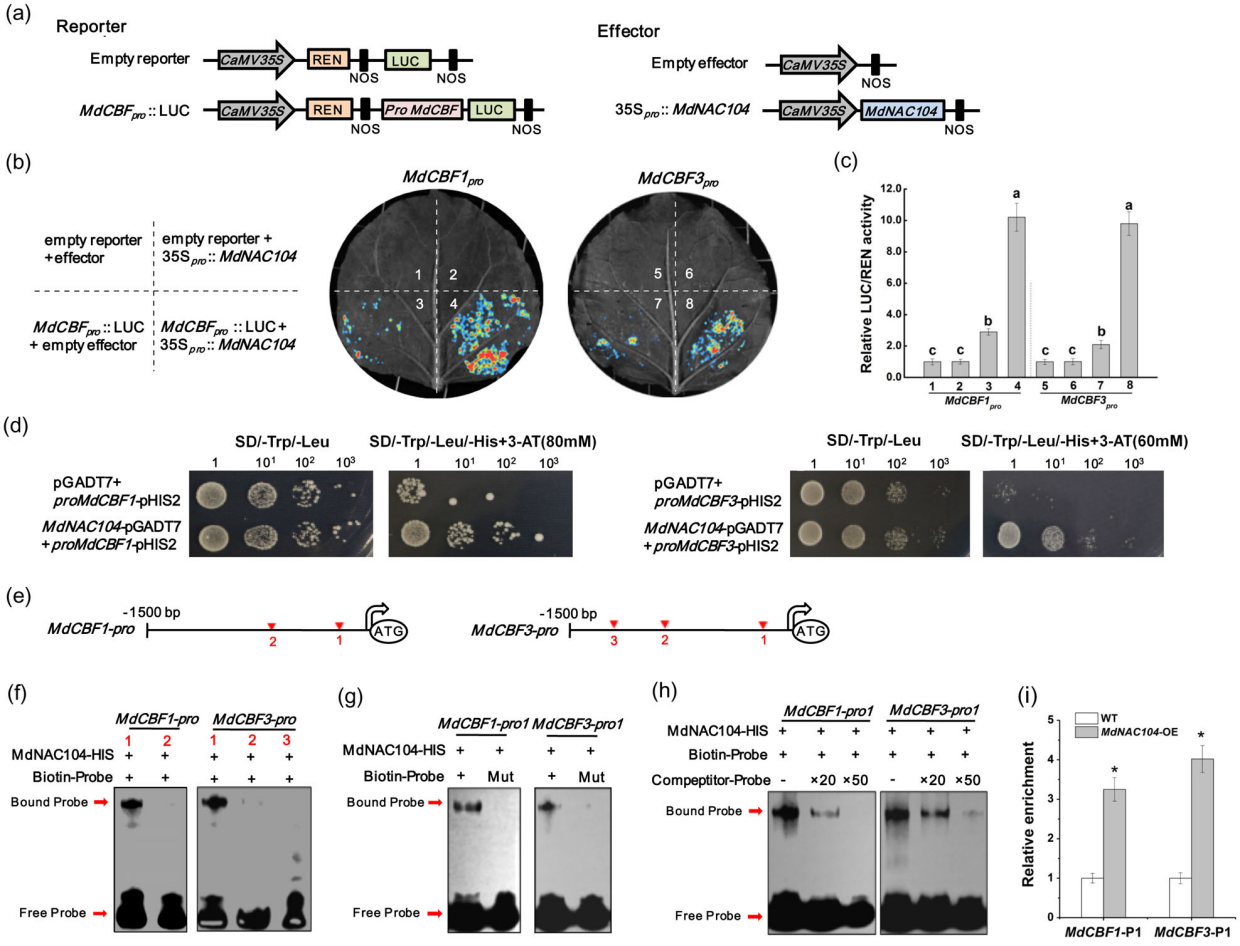
MdNAC104 directly binds to the *MdCBF1*/*MdCBF3* promoters and promotes their expression. (a) Schematic diagram of the reporter and effector vectors. (b) Fluorescence observations of the dual‐luciferase assay. (c) Relative LUC/REN activity. Error bars indicate the SE of three biological replicates. Different letters represent significant differences (*P* < 0.05) (d) Y1H was used to identify the binding ability of the MdNAC104 protein to the *MdCBF1* and *MdCBF3* promoters. (e) Diagram of the putative NAC TF binding sites in the *MdCBF1* and *MdCBF3* promoters. (f) EMSA showing the binding of MdNAC104 to the *MdCBF1* and *MdCBF3* promoters. Numbers represent the putative NAC binding site in (e). (g–h) EMSAs with mutant probes (g) and competing probes (h) were performed to verify the promoter binding specificity of MdNAC104. Mut, mutant probes. ‘+’, presence. ‘−’, absence. 20× and 50× represent the rates of the competitor probes. (i) ChIP‐qPCR analysis indicates the binding of MdNAC104 to the *MdCBF1/MdCBF3* promoters.

Some NAC TFs bind to *CBF* promoters and regulate their expression; thus, participating in the regulation of the cold stress response (An *et al*., 2018a; Hao *et al*., 2011; Shan *et al*., 2014). As overexpressing *MdNAC104* significantly increased the transcription levels of *MdCBF1/3* and their downstream cold‐responsive genes (Figure 3) (Yang *et al*., 2023), *MdCBF1/3* may be the target gene of MdNAC104. We cloned the *MdCBF1*/*MdCBF3* promoter sequences and identified the transcriptional activation ability of MdNAC104 on their promoters using the LUC/REN dual‐luciferase system. The *MdCBF1* and *MdCBF3* promoter sequences were inserted into the reporter vector, and the MdNAC104 CDS was cloned into the effector vector, respectively (Figure 4a). Then, these constructs were co‐expressed in tobacco leaves with specified combinations. After the cold treatment, the results of fluorescence observations and relative LUC/REN activity measurements indicated that co‐expressing the MdNAC104 protein significantly increased the transcriptional activity of the *MdCBF1* and *MdCBF3* promoters. These results indicate that MdNAC104 transcriptionally activated *MdCBF1*/*MdCBF3* at low temperatures.

Y1H assays were performed to identify the interaction between MdNAC104 and the *MdCBF1*/*MdCBF3* promoters. The yeast strains co‐transformed with the MdNAC104‐pGADT7 and *MdCBF1*‐pHIS2 (or *MdCBF3*‐pHIS2) vectors grew in the selection medium supplemented with 3‐AT, while the control strains transformed with the pGADT7 empty vectors did not (Figure 4d), indicating the binding ability of MdNAC104 on the *MdCBF1*/*MdCBF3* promoters. EMSA was conducted to further identify the promoter binding ability of MdNAC104. Promoter sequence analysis revealed that the *MdCBF1* and *MdCBF3* promoters contained two and three putative NAC TF binding elements, respectively (Figure 4e). Biotin‐labelled probes were synthesized according to the flanking sequences of these sites (Table S1). The EMSA results showed that the MdNAC104 protein bound to site 1 in the *MdCBF1* and *MdCBF3* promoters (Figure 4f). When the core recognition element sequence of the probe was mutated, the binding band disappeared (Figure 4g; Table S1). Furthermore, adding unlabelled competing probes significantly reduced the brightness of the bands, indicating the specificity of the MdNAC104 protein binding to these two sites (Figure 4h).

To determine the binding of MdNAC104 to the *MdCBF1*/*MdCBF3* promoters *in vivo*, *MdNAC104‐MYC* overexpressing transgenic apple calli were obtained (Figure S3a). The cold treatment (4 °C) had a significant inhibitory effect on growth of apple calli, which was relieved by overexpressing *MdNAC104*, and the fresh weights of transgenic calli were significantly higher than those of the wild type (Figure S3b,c). Subsequently, the calli treated at 4 °C for 6 h were used for ChIP‐qPCR analysis. The results demonstrated that MdNAC104 bound to site 1 of the *MdCBF1* and *MdCBF3* promoters *in vivo* (Figure 4i). These results suggest that MdNAC104 directly binds to the promoters of *MdCBF1* and *MdCBF3* to enhance their expression under cold stress.

### Transcriptome and metabolome analyses demonstrate that MdNAC104 enhances proline and anthocyanin biosynthesis under cold conditions

**Figure 5:**
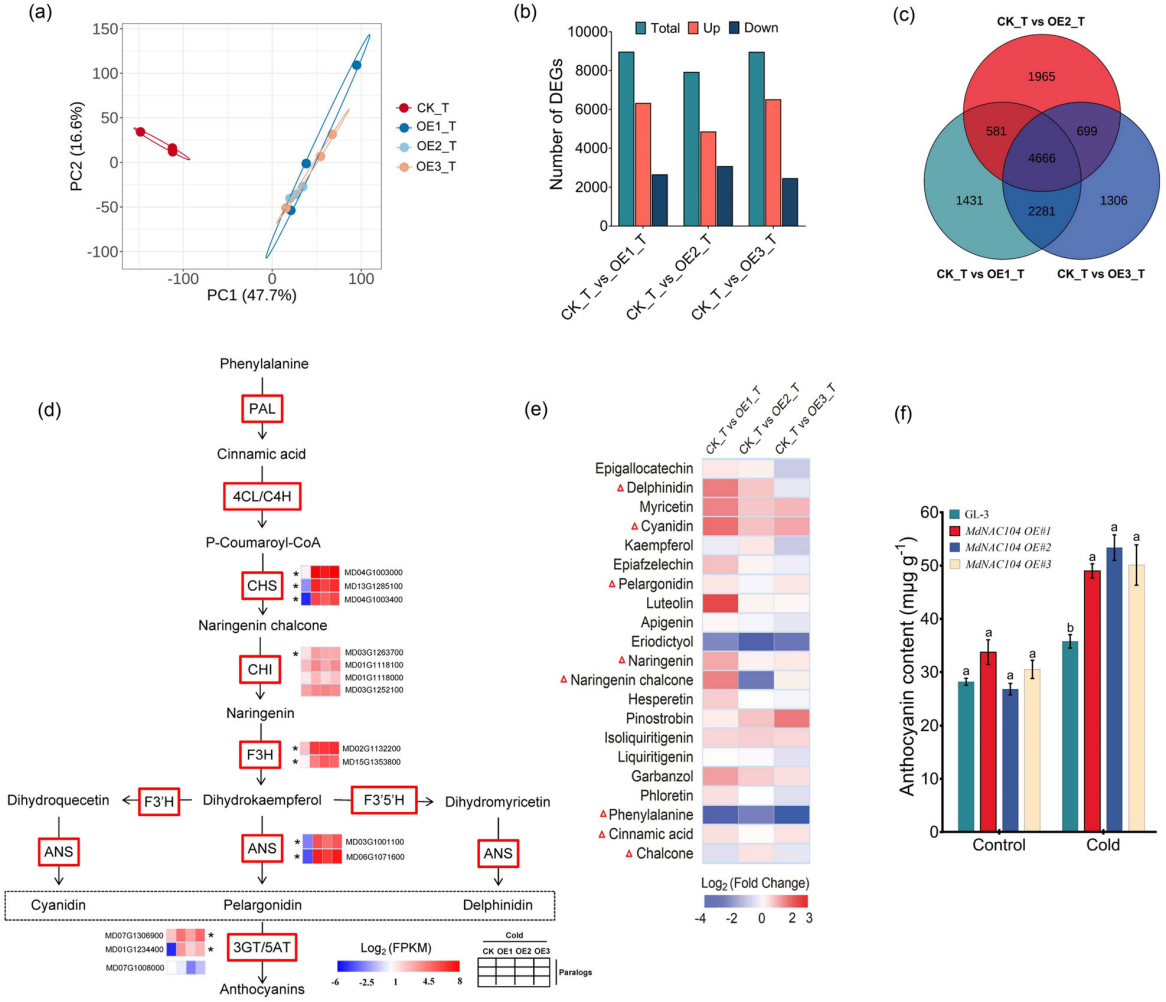
MdNAC104 positively regulates the expression of anthocyanin biosynthesis‐related genes and anthocyanin accumulation in apple leaves. (a) PCA analysis of the gene expression profile in apple leaves under cold treatment (T). (b) Statistics of the DEGs between *MdNAC104* transgenic (OE1, OE2 and OE3) and GL‐3 (CK) plants under the cold treatment. (c) Venn diagram analysis of the DEGs. (d) The expression patterns of the key genes involved in anthocyanin synthesis. *, DEGs shared among the three groups. (e) A comparative analysis of the levels of flavonoid‐related metabolites in the groups. Triangles indicate products of the anthocyanin metabolic pathway. (f) Anthocyanin content. The anthocyanin content of apple leaves was determined after a 30‐day 4 °C treatment. Error bars indicate the SE of three biological replicates. Different letters represent significant differences (*P* < 0.05).

To further elucidate the potential regulatory mechanism of *MdNAC104* in the cold response, the leaves of GL‐3 and *MdNAC104* transgenic apple plants were collected after an 8‐h treatment at 4 °C and analysed by RNA‐Seq and UPLC‐MS/MS metabolomics, respectively. Transcriptome sequencing of 12 samples (GL‐3 and three transgenic lines OE‐1, OE‐2 and OE‐3, with three biological replicates each) obtained 106.9 Gb of data, in which the clean data of each sample was >8.03 Gb, and the aligned reads were >92.5%. The GC contents of the 12 samples were 46.44%–47.15%, and the proportion of Q30 bases was >92.64%, indicating the high accuracy of the sequencing data (Table S4). Principal component analysis (PCA) analysis of the gene expression profile showed that the biological repeats of the same line clustered together well, and the transgenic lines and GL‐3 were distinguished (Figure 5a). This result indicates that the expression profiles of the transgenic and GL‐3 plants were significantly different under cold conditions.

By comparing the expression profiles between the three transgenic lines and the wild‐type, 8959 (including 6312 upregulated and 2647 downregulated), 7911 (including 4846 upregulated and 3065 downregulated) and 8952 (including 6504 upregulated and 2448 downregulated) DEGs were identified in the comparisons CK_T_vs._OE1_T, CK_T_vs._OE2_T and CK_T_vs._OE3_T, respectively (Figure 5b; Figure S4a). The KEGG pathway and GO enrichment analyses showed that these DEGs were significantly enriched in ‘plant hormone signal transduction’, ‘starch and sucrose metabolism’, ‘phenylpropane biosynthesis’ and ‘flavonoid biosynthesis’, among others (Figure S4b,c). Use of UPLC‐MS/MS metabolomics allowed us to identify 973 metabolites, with 42, 42 and 39 different metabolites identified between the three groups, respectively (Figure S5a,b). Functional enrichment analysis showed that the different metabolites were mainly enriched in pathways, such as ‘secondary metabolite biosynthesis’, ‘amino acid biosynthesis’, ‘ABC transporter’, ‘arginine and proline metabolism’, ‘phenylpropane biosynthesis’, ‘plant hormone biosynthesis’ and ‘flavonoid biosynthesis’ (Figure S5d). The transcriptome analysis revealed that the expression of several key genes in the proline synthesis pathway, such as *Mdami*, *MdspeD* and *MdALDH*, were significantly upregulated in transgenic plants (Figure S6a). The contents of corresponding metabolites, such as L‐glutamic acid, 4‐guanidinobutyric acid and S‐adenosylmethionine, also increased significantly (Figure S6a,b). These results, combined with the higher proline content in transgenic plants under cold conditions (Figure 1j), indicate that MdNAC104 may enhance apple cold tolerance by promoting proline synthesis and accumulation.

Anthocyanins, which are products of the flavonoid metabolic pathway, play an important role in resisting low‐temperature injury (Jiang *et al*., 2022). Venn diagram analysis of the DEGs showed that there were 4666 DEGs in all three groups (Figure 5c). Functional annotation revealed that several key genes in the anthocyanin biosynthetic pathway were included, and their expression levels were significantly upregulated in transgenic plants (Figure 5d; Table S5). The metabolomic analysis detected 21 flavonoid metabolic pathway products (Figure 5e). We analysed the expression patterns of the DEGs and the contents of related metabolites involved in the anthocyanin biosynthesis pathway in detail (Figure 5d,e). After the cold treatment, the expression levels of three *MdCHS* genes (*MdCHS‐a*, *MD04G1003000*; *MdCHS‐b*, *MD04G1003400*, and *MdCHS‐c*, *MD13G1285100*) were significantly upregulated in transgenic plants, and the contents of pinostrobin and isoliquiritigenin increased significantly; four *MdCHI* (*MdCHI‐a*, *MD03G1263700*; *MdCHI‐b*, *MD01G1118100*; *MdCHI‐c*, *MD01G1118000*; *MdCHI‐d*, *MD03G1252100*) genes, two *MdANS* (*MdANS‐a*, *MD03G1001100* and *MdANS‐b*, *MD06G1071600*) and two *MdF3H* (*MdF3H‐a*, *MD02G1132200* and *MdF3H‐b*, *MD15G1353800*) genes were significantly upregulated, and the contents of the corresponding metabolites, such as naringenin and pinostrobin, increased significantly (Figure 5d,e). Moreover, the anthocyanin content in the leaves of *MdNAC104* transgenic plants was significantly higher than that in GL‐3 plants after the cold treatment (Figure 5f). These results indicate that MdNAC104 may enhance the cold tolerance of apple plants by promoting anthocyanin synthesis and accumulation at low temperatures.

### MdNAC104 directly binds to the promoters of the anthocyanin synthesis‐related genes and promotes their expression

NAC TFs in apple positively regulate the expression of multiple genes in the flavonoid pathway and anthocyanin accumulation by interacting with several TFs (Sun *et al*., 2019; Zhang *et al*., 2020). To explore the mechanism of how MdNAC104 promotes the expression of genes within the anthocyanin synthesis pathway, we first identified whether MdNAC104 interacts with the classical TFs that regulate anthocyanin accumulation in apple, including MdMYB1, MdbHLH3, MdbHLH33, MdMYC2 and MdHY5. Unfortunately, the results of Y2H experiments showed that none of these TFs interacted with the MdNAC104 protein (Figure S7).

**Figure 6:**
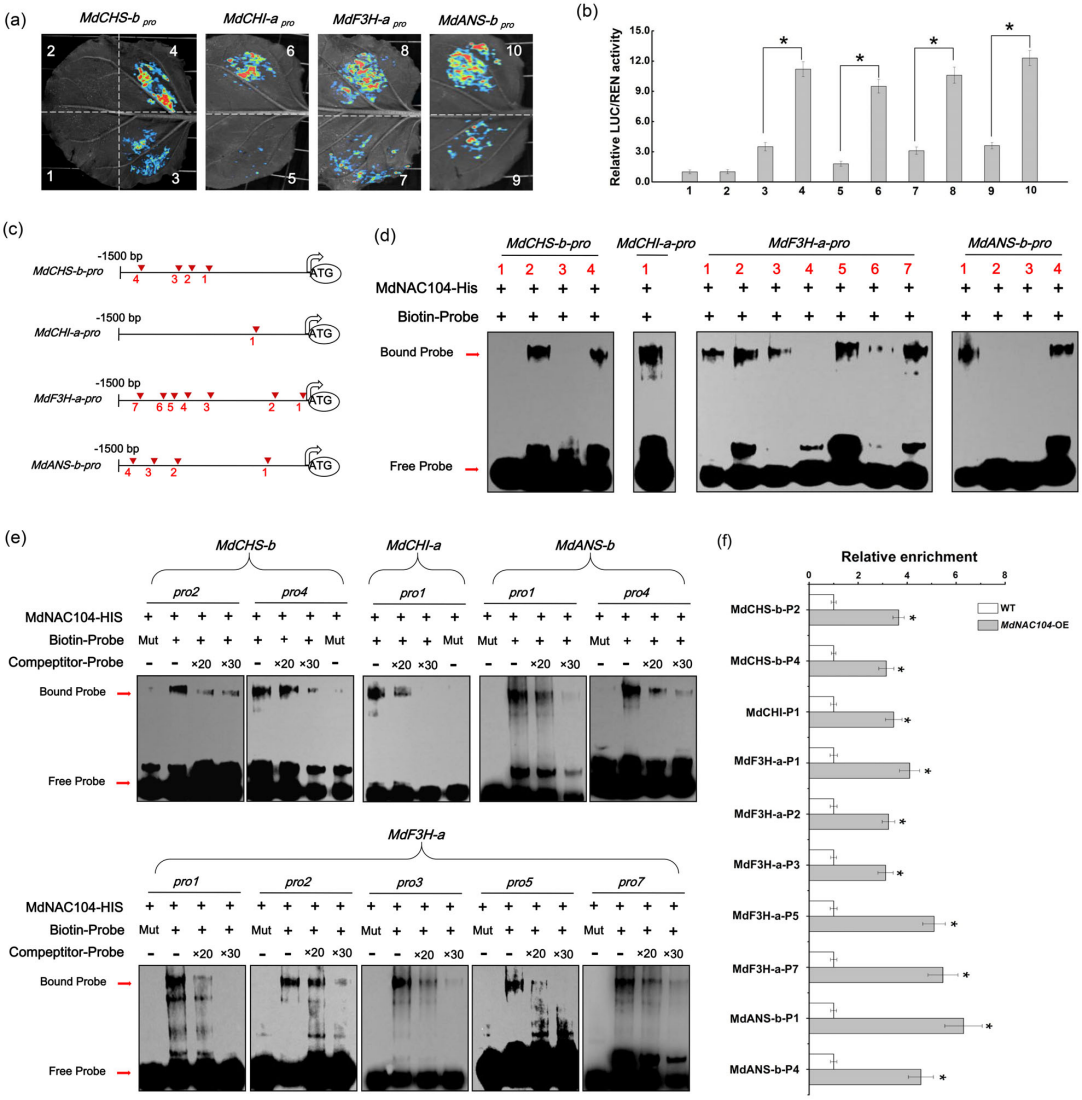
MdNAC104 directly binds to the *MdCHS‐b*, *MdCHI‐a*, *MdF3H‐a* and *MdANS‐b* promoters and activates their expression. (a, b) LUC/REN dual‐luciferase assays showing that MdNAC104 promotes the transcriptional activity of the *MdCHS‐b*, *MdCHI‐a*, *MdF3H‐a* and *MdANS‐b* promoters. (a) Fluorescence observations. (b) LUC/REN relative activity determination. The promoter fragments of the target genes were inserted into the pGreenII 0800‐Luc reporter vector, and the *MdNAC104* CDS was cloned into the pGreenII 62‐SK effector vector. 1: Empty reporter and effector, 2: empty reporter+35S_pro_::MdNAC104, 3: proMdCHS‐b::LUC+empty effector, 4: proMdCHS‐b::LUC+35S_pro_::MdNAC104, 5: proMdCHI‐a::LUC+empty effector, 6: proMdCHI‐a::LUC+35S_pro_::MdNAC104, 7: proMdF3H‐a::LUC+empty effector, 8: proMdF3H‐a::LUC+35S_pro_::MdNAC104, 9: proMdANS‐b::LUC+empty effector, 10: proMdANS‐b::LUC+35S_pro_::MdNAC104. Error bars indicate the SE of three biological replicates. One‐way ANOVA (Tukey's test) was performed, and significant differences are indicated by: *P < 0.05. (c) Diagram of the putative NAC TF binding sites in the *MdCHS‐b*, *MdCHI‐a*, *MdF3H‐a* and *MdANS‐b* promoters. (d) EMSA demonstrating that MdNAC104 binds to the *MdCHS‐b*, *MdCHI‐a*, *MdF3H‐a* and *MdANS‐b* promoters. (e) EMSAs were performed with the mutant and competing probes to verify the binding specificity of MdNAC104. ‘+’, presence. ‘−’, absence. 20× and 30× represent the rates of competitor probes. (f) ChIP‐qPCR analysis indicates the binding of MdNAC104 to the *MdCHS‐b*, *MdCHI‐a*, *MdF3H‐a* and *MdANS‐b* promoters.

Based on the transcriptome and metabolome results, we speculated whether MdNAC104 directly regulated the expression of anthocyanin synthesis‐related genes. Four anthocyanin synthesis‐related genes, such as *MdCHS‐b*, *MdCHI‐a*, *MdF3H‐a* and *MdANS‐b*, whose expression levels were significantly upregulated in the transcriptome (Table S5), were selected for cloning the promoter. Then, the transcriptional activation ability of MdNAC104 on these promoters was identified by the LUC/REN dual‐luciferase system. The results showed that co‐expression of the MdNAC104 protein significantly increased the transcriptional activity of these promoters under cold conditions (Figure 6a,b). Sequences analysis revealed the presence of several putative NAC TF binding elements in the promoters of these genes (Figure 6c). The EMSA results showed that the MdNAC104‐His protein bound directly to the second and fourth sites of the *MdCHS‐b* promoter, the first site of the *MdCHI‐a* promoter, the first, second, third, fifth and seventh sites of the *MdF3H‐b* promoter, and the first and fourth sites of the *MdANS‐b* promoter (Figure 6d). In contrast, no binding was observed when the His protein was added to the EMSA (Figure S8). The EMSA with the addition of mutant and competing probes further demonstrated the binding specificity of MdNAC104 to these sites (Figure 6e). In addition, ChIP‐qPCR analyses were performed to identify the binding ability of MdNAC104 to these sites *in vivo* (Figure 6f). The results showed that MdNAC104 directly bound to the promoters of these anthocyanin synthesis‐related genes to promote their expression under cold conditions, which synergistically promoting anthocyanin accumulation and enhancing cold tolerance of apple plants.

### MdNAC104 directly binds to the promoters of the antioxidant enzyme‐encoding genes *MdFSD2* and *MdPRXR1.1* and promotes their expression

**Figure 7:**
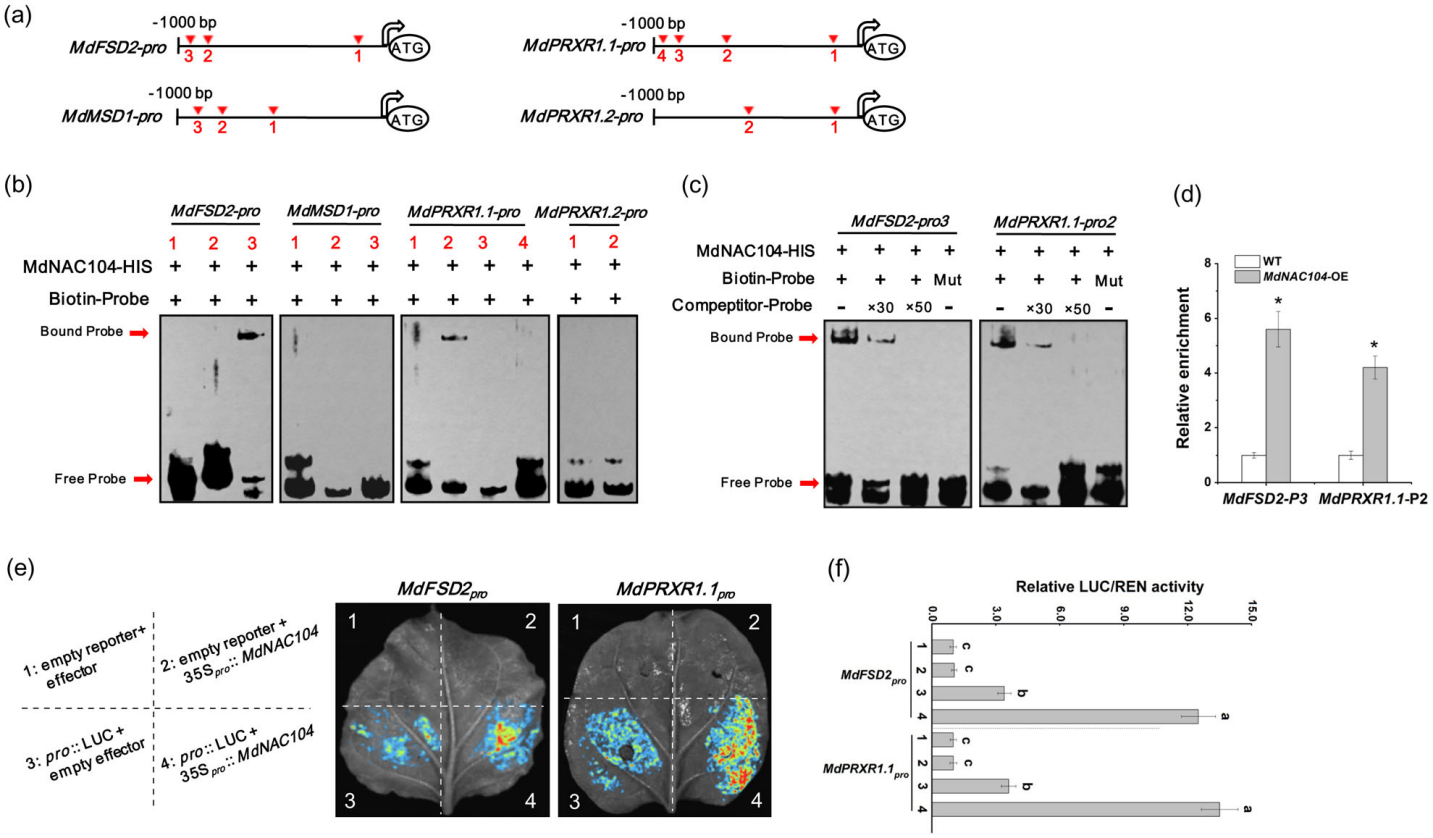
MdNAC104 directly binds to the *MdFSD2* and *MdPRXR1.1* promoters and activates their expression. (a) Diagram of the putative NAC TF binding sites in the *MdFSD2*, *MdMSD1*, *MdPRXR1.1* and *MdPRXR1.2* promoters. (b) EMSA demonstrating that MdNAC104 binds to the *MdFSD2* and *MdPRXR1.1*. promoters. (c) EMSAs were performed with the mutant and competing probes to verify the binding specificity of the MdNAC104 protein. ‘+’, Presence. ‘−’, Absence. 30× and 50× represent the rates of competitor probes. (d) ChIP‐qPCR analysis indicates the binding of MdNAC104 to the *MdFSD2* and *MdPRXR1.1* promoters. (e, f) Fluorescence observations (e) and relative LUC/REN activity measurements (f) in the LUC/REN dual‐luciferase assays. Error bars indicate the SE of three biological replicates. Different letters represent significant differences (*P* < 0.05).

Overexpressing *MdNAC104* significantly enhanced the activities of antioxidant enzymes, thereby promoting ROS scavenging under cold stress (Figure 2). We assessed the transcript levels of all genes encoding SOD, POD and CAT. The expression levels of most SOD and POD encoding genes in the *MdNAC104* transgenic lines were significantly upregulated compared to those in GL‐3 plants. Among these genes, the expression levels of two SOD encoding genes (*MD07G1240700*, *MD01G1164700*) and two POD (*MD10G1321200*, *MD05G1345800*) genes were significantly upregulated in all groups, with the highest upregulation ratio (Table S6). Based on the blastp analysis, these four genes were renamed *MdFSD2* (*MD07G1240700*), *MdMSD1* (*MD01G1164700*), *MdPRXR1.1* (*MD10G1321200*) and *MdPRXR1.2* (*MD05G1345800*), following their homologous genes in *Arabidopsis*, respectively. The promoter sequences of these genes (1000 bp before ATG) were extracted from the apple genome. Multiple NAC TF binding sites were detected in their promoters (Figure 7a).

To identify whether MdNAC104 directly binds to the promoters of these antioxidant enzyme‐encoding genes, biotin‐labelled probes were synthesized based on the sequences around these putative NAC TF binding sites, and EMSAs were carried out. The EMSA results indicated that the MdNAC104 protein bound directly to the third site of the *MdFSD2* promoter and the second site of the *MdPRXR1.1* promoter (Figure 7b). By adding the mutation and competition probes, the EMSA showed that MdNAC104 specifically bound to these sites (Figure 7c). In addition, ChIP‐qPCR experiments were performed using *MdNAC104‐MYC* transgenic calli, and the results further demonstrated the binding ability of MdNAC104 to these sites *in vivo* (Figure S7d). LUC/REN assays were performed to further identify the transcriptional activation activity of MdNAC104 on the *MdFSD2* and *MdPRXR1.1* promoters. The results showed that co‐expressing the MdNAC104 protein significantly enhanced the transcriptional activities of the *MdFSD2* and *MdPRXR1.1* promoters under cold conditions (Figure 7e,f). *MdFSD2* and *MdPRXR1.1* were also the most upregulated genes among the SOD and POD encoding genes, respectively (Table S6). These results show that MdNAC104 increased SOD and POD activities by promoting *MdFSD2* and *MdPRXR1.1* expression; thus, promoting the scavenging of excess ROS and enhancing the cold tolerance of apple plants.

## Discussion

Temperature is one of the main environmental factor affecting crop yield and quality as well as geographical distribution. Cold stress usually retards the growth of plants and causes gradual leaf browning and wilting, tissue softening and other symptoms (Chinnusamy *et al*., 2007; Feng *et al*., 2013; Hu *et al*., 2019; Xie *et al*., 2018). An increase in the frequency of extremely low‐temperature weather events has been observed with global climate change, which has impacted the development of the fruit tree industry. For example, apple production in China can be greatly impacted by cold stress, especially early spring chilling and late spring frosts (An *et al*., 2020; Feng *et al*., 2012). Studying the function and regulatory mechanism of key genes in the cold response is important for the stress resistance breeding of fruit crops. NAC TFs play an important role in the regulation of plant growth and the stress response (Srivastava *et al*., 2022). However, the mechanisms underlying NAC‐mediated cold tolerance are not well understood, particularly in fruit crops. Here, our results show enhanced freezing tolerance in transgenic apple plants overexpressing *MdNAC104*. The plants exhibited less leaf damage, a lower REL and a higher survival rate (Figure 1), suggesting a positive role for MdNAC104 in the apple cold response.

### *MdCBF1* and *MdCBF3* participate in MdNAC104‐mediated cold stress response in apple

The CBF‐COR pathway is a well‐known cold signalling pathway in plants. Once exposed to low temperatures, CBFs are rapidly activated by cold signalling to promote the expression of the downstream cold‐responsive genes, thus positively regulating cold tolerance (Ding and Yang, 2022; Kidokoro *et al*., 2022; Shi *et al*., 2018). CBF1 and CBF3 are two representative factors in the *Arabidopsis* CBF family that positively regulate the cold response (Dong *et al*., 2020; Kidokoro *et al*., 2022). *MdCBF1* and *MdCBF3* also play important roles in modulating cold tolerance in apples (An *et al*., 2017; Yang *et al*., 2023). Research has demonstrated that several NACs in different species play a role in the cold response by changing the expression of *CBF* genes, such as in tomatoes (Wang *et al*., 2018a), apples (An *et al*., 2018a), pear (Jin *et al*., 2017) and banana (Shan *et al*., 2014). Due to the significantly upregulated expression of *MdCBF1*/*MdCBF3* and downstream cold‐responsive genes in *MdNAC104* transgenic plants under cold stress (Figure 3), and the multiple putative NAC TF binding sites in the *MdCBF1/MdCBF3* promoters (Figure 4e), we speculate that MdNAC104 may be involved in the regulation of cold stress tolerance by directly activating *MdCBF1/MdCBF3* expression. Promoter binding and transcriptional activation analyses showed that MdNAC104 directly bound to the *MdCBF1*/*MdCBF3* promoters and enhanced their expression, thereby activating downstream cold signalling in apple plants (Figures 3 and 4). These results indicate that MdNAC104 regulates cold tolerance in apple plants via the CBF‐dependent pathway.

### MdNAC104 enhances apple cold tolerance by promoting the accumulation of osmoregulatory substances

Plants have evolved a series of complex physiological responses to protect themselves from freezing damage, including improved membrane stability and increased accumulation of osmoregulatory substances (Guo *et al*., 2018). The accumulation of osmoregulatory substances, such as soluble sugars, betaine and proline, helps to maintain high cell osmotic pressure, thereby stabilizing membrane structure, maintaining normal cell functions and improving cold tolerance (Feng *et al*., 2013). The cold treatments in apples significantly increased the accumulation of soluble sugars, soluble proteins and proline in leaves (Figure 1h‐j) (Dong *et al*., 2011; Xu *et al*., 2022a). Furthermore, the accumulation of these osmoregulatory substances in *MdNAC104*‐overexpressing transgenic apple plants was significantly higher than that in GL‐3 plants (Figure 1h–j), indicating that MdNAC104 enhanced apple cold tolerance by promoting the accumulation of these osmoregulatory substances. Transcriptome and metabolome analyses showed that the expression levels of several proline synthetic genes were significantly upregulated, and the contents of related metabolites also significantly increased in transgenic plants compared with GL‐3 plants (Figure S6). These results reveal that MdNAC104 increased the accumulation of proline by promoting proline synthesis in response to cold stress. The results of the KEGG pathway enrichment analysis of the DEGs and metabolites indicated that this hypothesis also likely applies to soluble sugars and soluble proteins (Figures S4 and S5).

### MdNAC104 enhances apple cold tolerance by increasing the activities of the antioxidant enzymes

Cold stress causes excess accumulation of ROS in plants, leading to oxidative stress damage (Smirnoff and Arnaud, 2019; Wang *et al*., 2018c; Yang *et al*., 2022a,b). Excess ROS are removed by a series of enzymatic scavenging systems and non‐enzymatic antioxidants (Waszczak *et al*., 2018). SOD, POD, and CAT are the main antioxidant enzymes, which promote ROS scavenging under various abiotic stress conditions, such as drought (Laxa *et al*., 2019), cold (Chan *et al*., 2016; Hu *et al*., 2019) and high salinity (Rubio *et al*., 2009), thus inhibiting the excessive accumulation of ROS caused by stress (Wang *et al*., 2018c). The accumulation of H_2_O_2_ and O_2_^−^ in the *MdNAC104* transgenic lines under cold stress was significantly lower than that of the GL‐3 plants, while SOD and POD activities were significantly higher than those in GL‐3 plants (Figure 2). These results suggest that MdNAC104 promotes the removal of excess ROS by increasing the activity of antioxidant enzymes under cold stress. To verify this hypothesis, the expression levels of all genes that were annotated as antioxidant enzyme (SODs, PODs and CATs)‐encoding genes in the transcriptome were analysed. The expression levels of multiple SOD and POD encoding genes were significantly higher in the transgenic plants than in the GL‐3 plants (Table S6). Additionally, MdNAC104 specifically bound to the *MdFSD2* and *MdPRXR1.1* promoters and activated their expression (Figure 7; Table S6). These results indicate that MdNAC104 increased SOD and POD activities by promoting, at least in part, the transcription of *MdFSD2* and *MdPRXR1.1*. Additional studies are needed to explore whether MdNAC104 directly regulates the expression of other SOD and POD encoding genes. No significant difference in the expression of CAT encoding genes was detected between *MdNAC104* transgenic and GL‐3 plants (Table S6), and the difference in CAT activity was not as significant as that of SOD and POD (Figure 2), indicating that *MdCATs* are not target genes of MdNAC104.

### MdNAC104 enhances apple cold tolerance by promoting the accumulation of anthocyanins

The non‐enzymatic antioxidants responsible for ROS scavenging include carotenoids, tocopherols and flavonoids (Aslam *et al*., 2022; Mittler, 2002; Mittler *et al*., 2022). Anthocyanins are important flavonoid metabolites, which act as ROS scavengers to protect against oxidative stress (Jin *et al*., 2022; Takos *et al*., 2006; Van Den Ende and El‐Esawe, 2014). A positive correlation has been demonstrated between anthocyanin accumulation and cold tolerance in many plant species (Ahmed *et al*., 2015; Schulz *et al*., 2016; Zhang *et al*., 2019). MdNAC52 and MdNAC42 in apples promote anthocyanin accumulation in response to light and UV‐B irradiation, respectively (Sun *et al*., 2019; Zhang *et al*., 2020). Nevertheless, it remains unclear whether NAC TFs regulate apple cold tolerance by regulating anthocyanin accumulation. The transcriptome analysis showed that the expression levels of several key genes in the anthocyanin synthesis pathway were significantly upregulated in *MdNAC104* transgenic plants (Figure 5d), and the contents of corresponding metabolites and anthocyanin also increased significantly (Figure 5e,f). These results indicate that MdNAC104 promotes anthocyanin accumulation under cold conditions by promoting the expression of anthocyanin synthesis‐related genes. Therefore, the four genes, such as *MdCHS‐b*, *MdCHI‐a*, *MdF3H‐a* and *MdANS‐b*, which were most upregulated in the anthocyanin synthesis pathway were selected for promoter binding and transcriptional activation analyses (Table S5). The results demonstrated that MdNAC104 directly bound to the promoters of these genes and promoted their expression (Figure 6; Figure S3), indicating that MdNAC104 enhances apple cold tolerance by promoting the accumulation of anthocyanins. Moreover, the direct transcriptional activation effect of MdNAC104 on multiple genes in the anthocyanin synthesis pathway suggests that it should be a key positive regulator of anthocyanin accumulation in apples in response to low temperature, similar to MdMYB1, MdMYB10 and MdbHLH3 (Espley *et al*., 2007; Takos *et al*., 2006; Telias *et al*., 2011; Xie *et al*., 2012).

**Figure 8:**
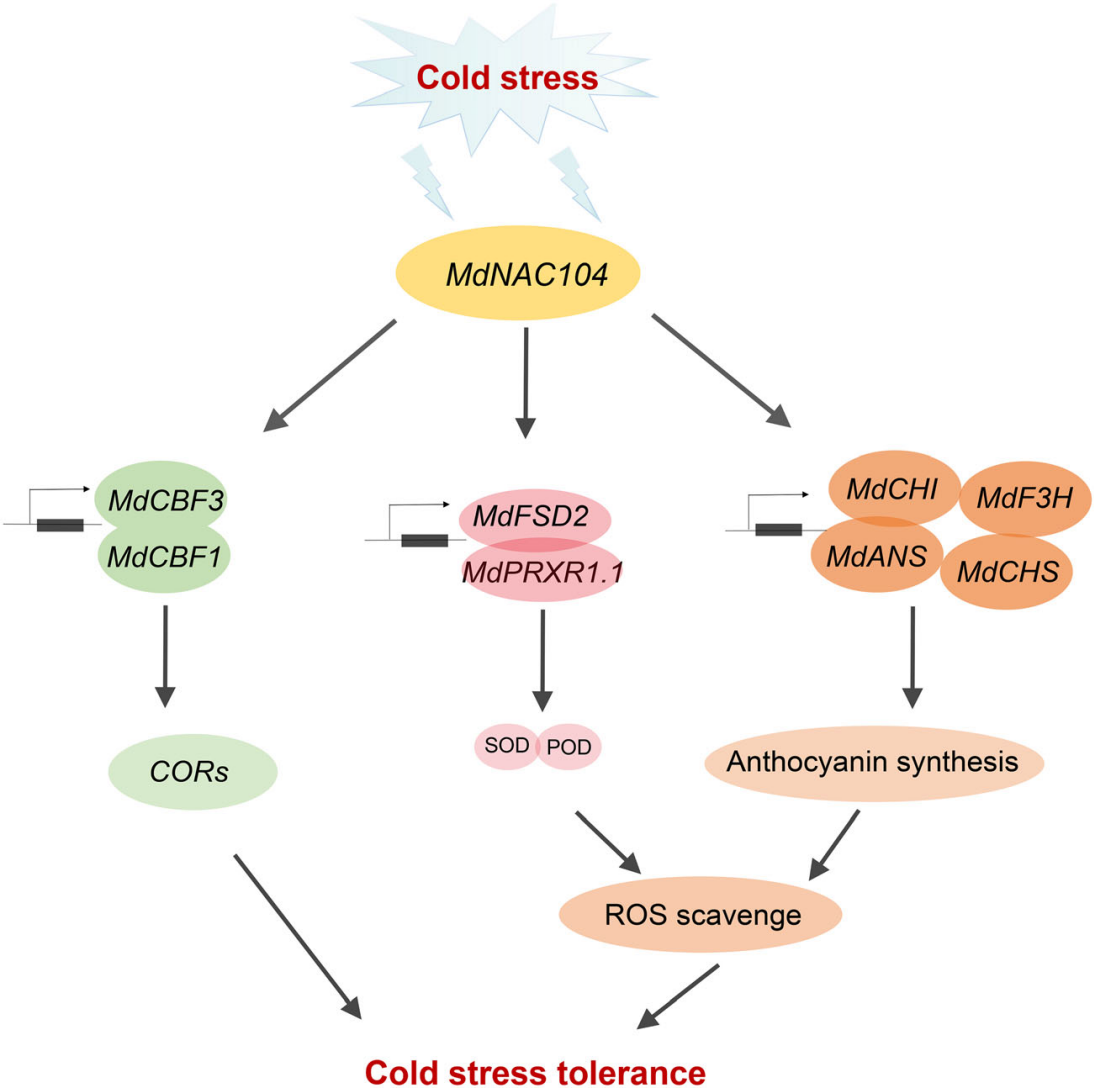
Working model showing the roles of MdNAC104 in cold stress signalling. MdNAC104 is rapidly activated upon exposure to cold stress. On the one hand, MdNAC104 positively regulates cold signalling in apple plants by directly activating *MdCBF1/MdCBF3* expression. On the other hand, MdNAC104 promotes anthocyanin accumulation and antioxidant enzyme activity by activating the expression of anthocyanin synthesis‐related genes and antioxidant enzyme‐encoding genes, respectively; thus, inhibiting excess accumulation of ROS caused by cold stress and enhancing the cold tolerance of apple plants.

Based on preceding studies, we propose a regulatory model by which MdNAC104 regulates cold tolerance in apple plants through CBF‐dependent and CBF‐independent pathways (Figure 8). MdNAC104 is activated rapidly upon exposure to cold stress. It directly binds to the *MdCBF1*/*MdCBF3* promoters to enhance their expression, thereby positively regulating cold tolerance in apple plants through the CBF‐COR pathway. MdNAC104 promoted anthocyanin accumulation and antioxidant enzyme activity by activating the expression of anthocyanin synthesis‐related genes (*MdCHS‐b*, *MdCHI‐a*, *MdF3H‐a* and *MdANS‐b*) and antioxidant enzyme‐encoding genes (*MdFSD2* and *MdPRXR1.1*) to inhibit the excess accumulation of ROS and enhance cold tolerance. The multiple roles of MdNAC104 in different pathways suggest that it is a key positive regulator in the apple cold stress response.

## Experimental procedures

### Plant materials

The *MdNAC104*‐overexpressing transgenic apple plants (OE#1, OE#2, and OE#3) utilized in this experiment were procured from our previous research (Jia *et al*., 2018). *MdNAC104‐MYC* transgenic apple calli (‘Orin’) were obtained by the *Agrobacterium tumefaciens* EHA105‐mediated transgenic method (Xie *et al*., 2012; Yang *et al*., 2021), using the pCambia35S‐4MYC‐3FLAG expression vector. The primers used to construct the vector are listed in Table S1.

### Freezing treatment

Tissue‐cultured *MdNAC104* transgenic and GL‐3 apple plants were rooted in rooting medium and then transplanted into pots (9 × 9 × 9 cm) containing a mixture of nutrient soil and Perlite (1:1, v/v) for 45 days of growth under a long‐day photoperiod (LD, 16‐h light/8‐h dark; 24 °C), with the daytime period set between 6:00 am and 22:00 pm. Subsequently, plants with consistent growth were selected and divided into a non‐cold‐acclimated treatment group (NA) and a cold‐acclimated group (CA). Each group contained three biological replicates, with 20 plants per line (OE#1, OE#2, OE#3 and GL‐3) in each replicate. The freezing treatments were carried out referring to the methods of Xie *et al*. (2018) and Yang *et al*. (2023) with some modifications.

The apple plants in the NA group were placed in a vertical cold light source incubator (photon flux density about 80 μmol/m^2^/s) and treated at −5 °C for 5 h, while the plants in the CA group were first acclimated to 4 °C for 72 h (LD) and then treated at −7 °C for 6 h (photon flux density about 80 μmol/m^2^/s). The freezing treatments started at 9:00 am and were carried out using a step‐by‐step cooling method, starting from 0 °C and decreasing the temperature at a rate of 2 °C/h until the final temperature, then maintaining the temperature for a specified time period. After the freezing treatment, the plants were directly transferred to 4 °C and held in the dark for 12 h of recovery. Then the plants were transferred to normal conditions (LD, 24 °C) for culture. After 2 days of culture, the phenotypes of the plants were determined and photographed. The survival rate of each line was determined after 15 days of culture under normal conditions (LD, 24 °C).

### Chilling treatment

Plants that cultured in pots for 45 days under LD condition (similar to those used for freezing treatments) were selected for chilling treatments. *MdNAC104* transgenic and GL‐3 plants with consistent growth were selected and treated at 4 °C for 8 h (photon flux density about 80 μmol/m^2^/s). Three biological replicates were performed, with 20 seedlings were in each line in each replicate.

Wild‐type and transgenic calli with consistent growth were treated at 4 °C under a dark condition for 20 days, then photographs were taken, and fresh weight was determined. Calli cultured under normal conditions (dark, 24 °C) were used as the control. Three biological replicates were performed, with five plates per replicate.

### Relative electrolyte leakage (REL) measurements

Plants that cultured in pots for 45 days under LD condition (similar to those used for freezing/chilling treatments) were used. The leaves from the same part (the fourth to sixth leaves from the top of the plants) of GL‐3 and transgenic plants grown under normal conditions were collected (the leaves of plants in the CA group were detached after cold acclimation). After washing with deionized water, two 1‐cm diameter leaf discs were collected with a hole punch and placed in a 10 mL ice‐water mixing test tube for treatment. Three independent biological replications were used, with each replicate containing 10 leaf discs from 5 plants per line. The low‐temperature water cycler uses a temperature‐variable programme as described by Xie *et al*. (2018). The temperature was decreased from 0 °C to −2 °C over 1 h and remained constant at −2 °C for 1 h; the temperature was dropped from −2 °C to −4 °C over 1 h and remained at −4 °C for 1 h; the temperature was dropped from −4 °C to −6 °C over 1 h and remained at −6 °C for 1 h; then was slowly increased to room temperature. The REL was measured according to the method described in previous study using the formula: REL = (D1−D0)/(D2−D0) × 100% (Yang *et al*., 2023), in which D1 indicates the conductivity before autoclaving, D2 indicates the conductivity after autoclaving, and D0 indicates the conductivity of deionized water. Leaves subjected to 0 °C were used as the control.

### Soluble sugar, soluble protein, proline and MDA content measurements

The contents of soluble sugars, proline, soluble proteins and MDA in the leaves of apple plants were determined in three biological replicates each using kits follow the manufacturer's instructions (Comin Biotechnology, Suzhou, China).

### H_2_O_2_ and O_2_ ^−^ staining and antioxidant enzyme activity measurements

The leaves of the plants were collected after an 8‐h treatment at 4 °C, and DAB and NBT staining were performed to observe the accumulation of H_2_O_2_ and O_2_^−^, respectively, according to the method described by Sun *et al*. (2018). Three biological replicates were used. Each staining treatment contained 10 leaves, and two leaves were collected from each plant. The H_2_O_2_ and O_2_^−^ contents and activities of the antioxidant enzymes SOD, POD and CAT were determined using kits following the manufacturer's instructions (Comin Biotechnology, Suzhou, China). Three biological replicates were used, with each replicate containing 10 leaves from five plants per line.

### Identification and quantification of metabolites

The leaves collected after the cold treatment (4 °C, 8 h) were lyophilized and ground in liquid nitrogen. A 10 mg portion of powder was weighed into a centrifuge tube, two steel balls were added, and 500 μL of extracting solution was added (methanol and water, volume ratio 3:1, pre‐cooled at −40 °C). The mixture was vortexed for 30 s and homogenized. The sonicated sample was placed in an ice‐water bath for 5 min and then overnight at 4 °C. The sample was centrifuged at 13 500 *g* for 15 min, and the supernatant was filtered through a 0.22 μm microporous membrane. The extract was diluted 10 times, mixed with 20 μL of each sample to form a quality control (QC) sample and stored at −80 °C until testing. The target compounds were separated by chromatography using a Waters ultra‐performance liquid chromatography column. An aqueous solution containing 0.1% formic acid was used as Phase A, and acetonitrile was used as Phase B. The column oven and autosampler temperatures were set to 40 and 4 °C, respectively. An injection volume of 2 μL was used. The column and liquid chromatography conditions are listed in Tables S2 and S3. A SCIEX 6500 QTRAP+ triple quadrupole mass spectrometer with an IonDrive Turbo V ESI ion source was used for mass spectrometry in multiple reaction monitoring mode. The ion source parameters were ion spray voltage: +5500/−4500 V; curtain gas: 35 psi; temperature: 400 °C; ion source gas 1:60 psi, ion source gas 2:60 psi; DP: ±100 V. The threshold values for identifying different metabolites were VIP > 1, fold change >1.2 or <0.8 and *P*‐value <0.05.

Functional pathway analysis of the different metabolites was performed using the KEGG pathway database. The R (V3.6.2) pheatmap package (The R Foundation for Statistical Computing, Vienna, Austria) was used to draw the clustering heatmap of the metabolites.

### RNA‐Seq analyses

After the 8‐h treatment at 4 °C, leaves were collected and ground into a powder in liquid nitrogen. Total RNA was extracted using an RNAprep pure Plant Kit (Tiangen, Beijing, China) according to the manufacturer's instructions. Transcriptome sequencing was conducted by the Genepioneer Biotechnologies Co., Ltd. (Nanjing, China). The sequencing data were aligned to the apple genome (GDDH13_1‐1; https://www.rosaceae.org/species/malus/all) using HISAT2 (v2.1.0) software, and transcriptome assembly and quantification were completed using stringtie software (v2.1.3b). FPKM (Fragments Per Kilobase of transcript per Million fragments mapped) was used to measure transcript levels or gene expression. The differentially expressed genes (DEGs) were identified using the following parameters: Log_2_FoldChange > =1, FDR < =0.05.

BLASTALL software (v2.2.26) was used for gene functional annotation based on the GO and KEGG databases. The R (v3.6.2) ggplot2 package was used to generate the DEG volcano maps. GraphPad Prism 7 (GraphPad Software, La Jolla, CA, USA) was employed to create the gene expression heatmaps.

### RNA extraction and real‐time fluorescence quantitative polymerase chain reaction (RT‐qPCR)

Leaves of apple plants from the chilling experiment were collected and used for RNA extraction. Total RNAs of the plant materials were isolated using an RNAprep pure Plant Kit (Tiangen, Beijing, China). The RNA was reverse transcribed using the PrimeScript RT Reagent Kit (TaKaRa, Shiga, Japan). RT‐qPCR analysis was performed as described previously (Mao *et al*., 2017), with *MdMDH* as the internal reference gene (Xie *et al*., 2018; Yang *et al*., 2023). Three biological replicates, each containing four technical replicates, were used. The primers used for the qRT‐PCR analysis are summarized in Table S1.

### GUS staining and activity

To construct the *MdNAC104pro*::GUS vector, the *MdNAC104* promoter fragment (1500 bp upstream of the start codon) was cloned into the pCambia1301‐GUS vector to replace the 35S promoter. The recombinant vector was injected into *N. benthamiana* leaves using the *Agrobacterium*‐mediated transformation method for transient expression (Yang *et al*., 2021). After the injection, the plants were cultured under normal conditions (25 °C; 16 h light/8 h dark) for 2 days, then subjected to a 6‐h cold treatment at 4 °C (photon flux density about 80 μmol/m^2^/s). Plants cultured under normal conditions were used as the control. Subsequently, the injected leaves were detached from the *N. benthamiana* plants and used for GUS staining and GUS activity measurements using kits following the manufacturer's instructions (Coolaber Science & Technology Co., Ltd., Beijing, China). Three biological replicates, with each replicate containing five plants were used. The primers used for constructing the vector are listed in Table S1.

### Yeast one‐hybrid (Y1H) assay

Y1H experiments were performed according to the instructions provided by Clontech (College Park, MD, USA) and as described previously (Yang *et al*., 2023). The *MdNAC104* CDS was cloned into the pGADT7 vector, and the promoter fragments of the target genes were inserted into the pHIS2 vector. These constructs were transformed into yeast strain Y187 at specified combinations and coated on SD/‐Trp/‐Leu medium. Individual clones were selected, and positive transformants were identified by genomic PCR. The positive transformants were inoculated on SD/‐Trp/‐Leu/‐His medium supplemented with different concentrations of 3‐amino‐1,2,4‐triazole (3‐AT). The growth of the strains was monitored and photographed after a 3‐day incubation at 28 °C. The yeast strain transformed with the empty pGADT7 vector was used as the negative control. The primers used to construct the vectors for the Y1H assay are summarized in Table S1.

### Dual‐luciferase assay

The promoter fragments of the target genes were inserted into the pGreenII 0800‐LUC reporter vector, and the *MdNAC104* CDS was cloned into the pGreenII 62‐SK effector vector, respectively. *Agrobacterium*‐mediated transformation allowed for the transient expression of a specified combinations of recombinant vectors in *N. benthamiana* leaves. After a 3‐d culture under normal conditions (25 °C, 16 h light/8 h dark), the plants were subjected to a 6‐h cold pretreatment at 4 °C. Subsequently, the injected *N. benthamiana* leaves were collected, and the fluorescence signals were captured using the Lumazone Pylon 2048B imaging system (Princeton, NJ, USA). The parts surrounding the injection site were collected to determine the relative LUC/REN activity using a detection kit (Promega, Madison, WI, USA).

### Electrophoretic mobility shift assay (EMSA)

The MdNAC104‐His fusion protein was expressed in *E. coli* BL21 and purified using a HIS purification column (Beyotime, Shanghai, China). EMSAs were performed using the LightShift Chemiluminescent EMSA kit (ThermoScientific Waltham, MA) following the manufacturer's instructions. The sequences of the various probes are summarized in Table S1.

### Chromatin immunoprecipitation (ChIP)‐qPCR assay

*MdNAC104‐MYC* transgenic calli were treated at 4 °C for 6 h, and then immersed in formaldehyde solution to promote protein‐DNA cross‐linking. After sonication, the calli were immunoprecipitated with an anti‐MYC antibody (Yeasen, Shanghai, China). The chromatin was purified, and the relative enrichment of the promoter fragment was detected by qPCR. Enrichment of the wild‐type callus served as the control (set to 1.0). Three independent biological replicates were used, each with four technical replicates. The ChIP‐qPCR primers are listed in Table S1.

### Statistical analysis

SPSS Statistics software (version 17; SPSS Inc., Chicago, IL) was used for the statistical analysis. Error bars indicate mean ± SE of three biological replicates. Significant differences were determined by one‐way analysis of variance (ANOVA) followed by Tukey's test. A *P*‐value <0.05 was considered significant.

#### Accession numbers

Apple sequence data can be found in the Genome Database for Rosaceae (GDR; https://www.rosaceae.org; *Malus × domestica* GDDH13 v1.1) with accession nos. MdNAC104 (MF401514.1, MD15G1415700), MdCBF1 (MD07G1262900), MdCBF3 (MD01G1196100), MdRD29A (MD01G1201000), MdCOR15A (MD09G1267600), MdCOR47 (MD08G1004500), MdKIN1 (MD09G1079600), MdCHS‐b (MD04G1003400), MdCHI‐a (MD03G1263700), MdF3H‐a (MD02G1132200), MdANS‐b (MD06G1071600), MdFSD2 (MD07G1240700), MdMSD1 (MD01G1164700), MdPRXR1.1 (MD10G1321200), MdPRXR1.2 (MD05G1345800). The transcriptome and metabolome data have been deposited at NCBI with the project ID PRJNA892233.

## Conflict of interest statement

None declared.

## Author contributions

C.M. and F.M. conceived the project. C.M., J.W., F.M. and K.M. designed the research plan. C.M., J.Y., D.J., Q.M., Q.G. and P.Y. carried out the experiments. C.M., J.Y., A.M., B.F., X.G. and K.M. analysed the data. C.M., J.Y., F.M. and K.M. wrote the manuscript. All authors read and approved of the content.

## Supporting information


**Figure S1** Sequence alignment between the MdNAC104 (MF401514.1; MD15G1415700) and MdNAC1 (MD10G1198400) proteins.
**Figure S2** Cold treatment increases transcription of the *MdNAC104* gene.
**Figure S3** ChIP‐qPCR analysis using *MdNAC104‐MYC* transgenic apple calli showing the promoter binding ability of MdNAC104 *in vivo*.
**Figure S4** Volcano plot (a), GO enrichment (b), and KEGG pathway enrichment (c) analyses of the DEGs identified in the transcriptome.
**Figure S5** Metabolome analysis of apple plants overexpressing *MdNAC104* at low temperature.
**Figure S6** MdNAC104 is involved in the regulation of proline biosynthesis.
**Figure S7** Protein interaction analysis between MdNAC104 and the transcription factors that promote anthocyanin accumulation in response to cold using the Y2H assay.
**Figure S8** EMSA demonstrates binding specificity between MdNAC104 and the biotin‐labelled probes.

**Table S1** The primers used in this study.
**Table S2** Liquid chromatography column conditions.
**Table S3** Liquid chromatography mobile phase conditions.
**Table S4** Summary of transcriptome sequencing data.
**Table S5** Expression and functional annotation of the anthocyanin synthesis‐related genes identified in the transcriptome.
**Table S6** Expression and functional annotation of the antioxidant enzyme‐encoding genes identified in the transcriptome.
