# Supplementary material for: MdNAC104 positively regulates apple cold tolerance via CBF‐dependent and CBF‐independent pathways
Source: Plant Biotechnol J. 2023 Jun 30;21(10):2057–73. doi: 10.1111/pbi.14112 (PMC10502760; doi:10.1111/pbi.14112)
Supplement: Supplementary file 1 — Figure S1 Sequence alignment between the MdNAC104 (MF401514.1; MD15G1415700) and MdNAC1 (MD10G1198400) proteins. Figure S2 Cold treatment increases transcription of the MdNAC104 gene. Figure S3 ChIP‐qPCR analysis using MdNAC104‐MYC transgenic apple calli showing the promoter binding ability of MdNAC104 in vivo. Figure S4 Volcano plot (a), GO enrichment (b), and KEGG pathway enrichment (c) analyses of the DEGs identified in the transcriptome. Figure S5 Metabolome analysis of apple plants overexpressing MdNAC104 at low temperature. Figure S6 MdNAC104 is involved in the regulation of proline biosynthesis. Figure S7 Protein interaction analysis between MdNAC104 and the transcription factors that promote anthocyanin accumulation in response to cold using the Y2H assay. Figure S8 EMSA demonstrates binding specificity between MdNAC104 and the biotin‐labelled probes. [file PBI-21-2057-s002.docx]

**
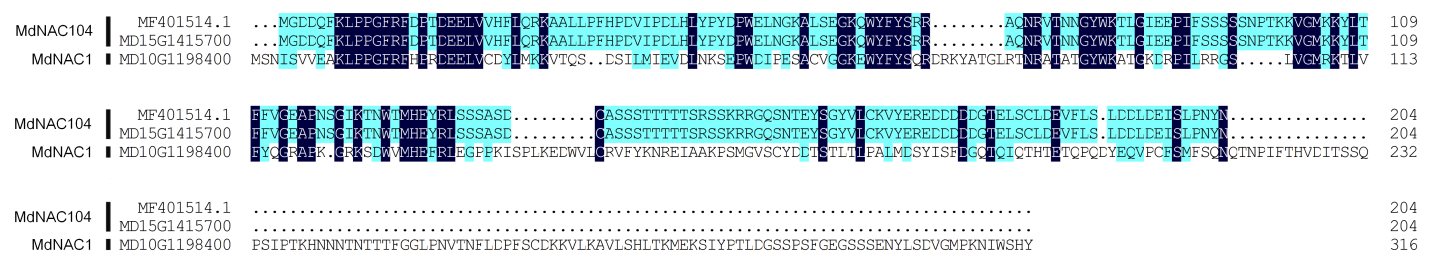
**

**Fig. S1** Sequence alignment between the MdNAC104 (MF401514.1; MD15G1415700) and MdNAC1 (MD10G1198400) proteins.

**
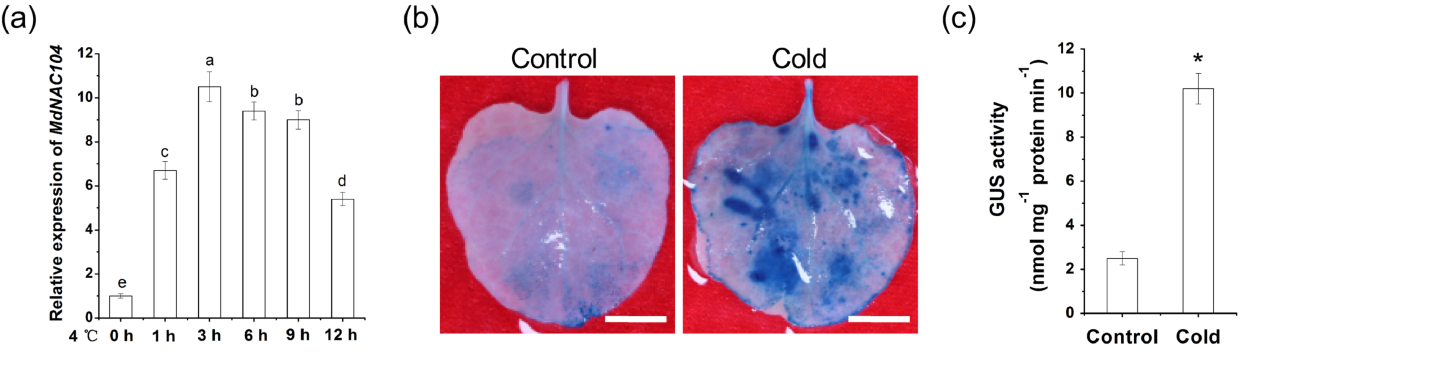
**

**Fig. S2** Cold treatment increases transcription of the *MdNAC104* gene. (a) RT-qPCR analysis of MdNAC104 expression under cold treatment. (b) GUS staining of *N. benthamiana* leaves expressing the *MdNAC104pro*::GUS vector. (c) GUS activity measurements.

**
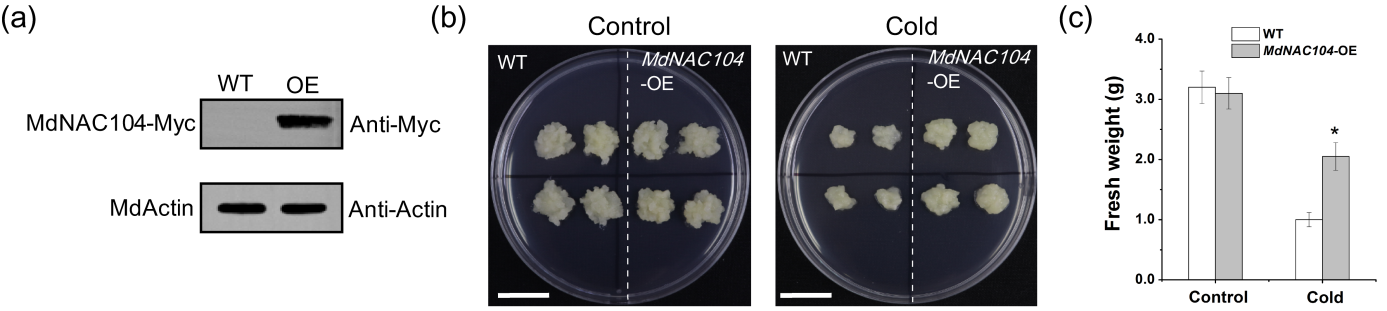
**

**Fig. S3** ChIP-qPCR analysis using *MdNAC104-MYC* transgenic apple calli showing the promoter binding ability of MdNAC104 *in vivo*. (a) Identifying *MdNAC104-MYC* transgenic apple calli by western blot using the anti-MYC antibody. (b) Phenotypes of the wild-type and *MdNAC104-MYC* transgenic calli grown under cold treatments. (c) Fresh weight measurements of the apple calli.

**
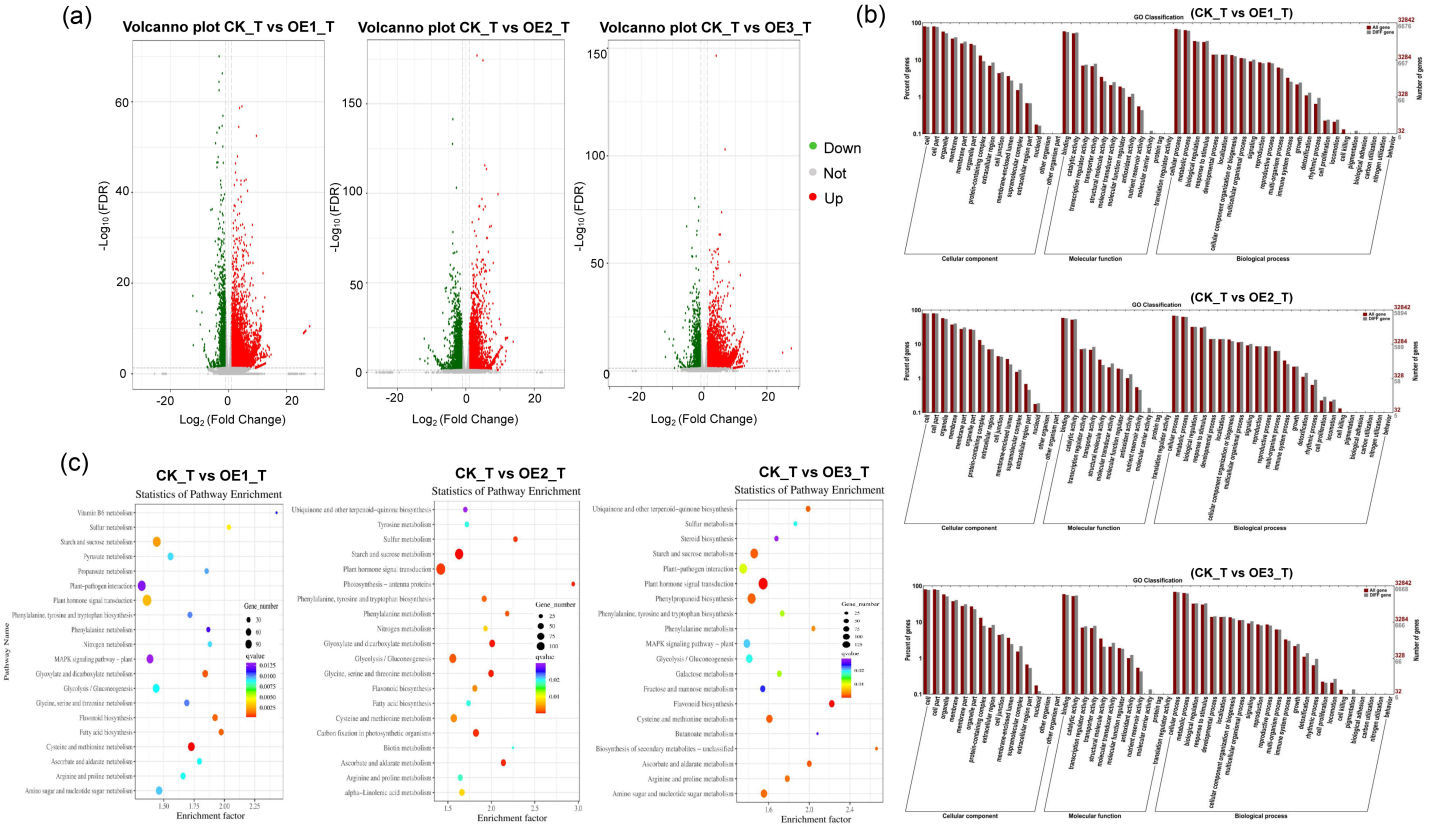
**

**Fig. S4** Volcano plot (a) and GO enrichment (b) and KEGG pathway enrichment analyses of the DEGs identified in the transcriptome.

**
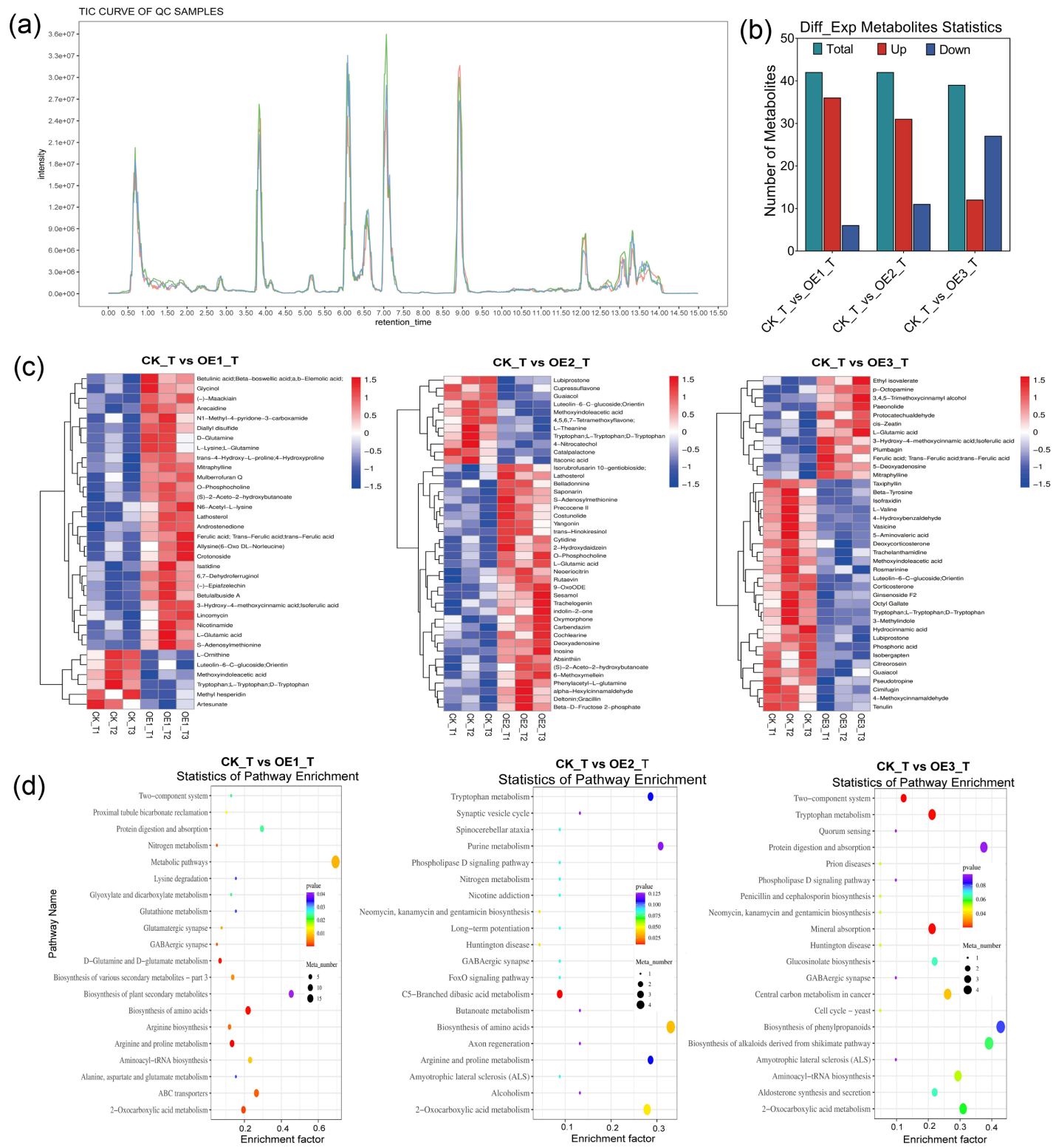
**

**Fig. S5** Metabolome analysis of apple plants overexpressing *MdNAC104* at low temperature. (a) TIC of the QC sample. (b) Summary of different metabolites in the groups. (c) Heat map of the relative contents of different metabolites. (d) KEGG pathway enrichment analysis of the different metabolites.

**
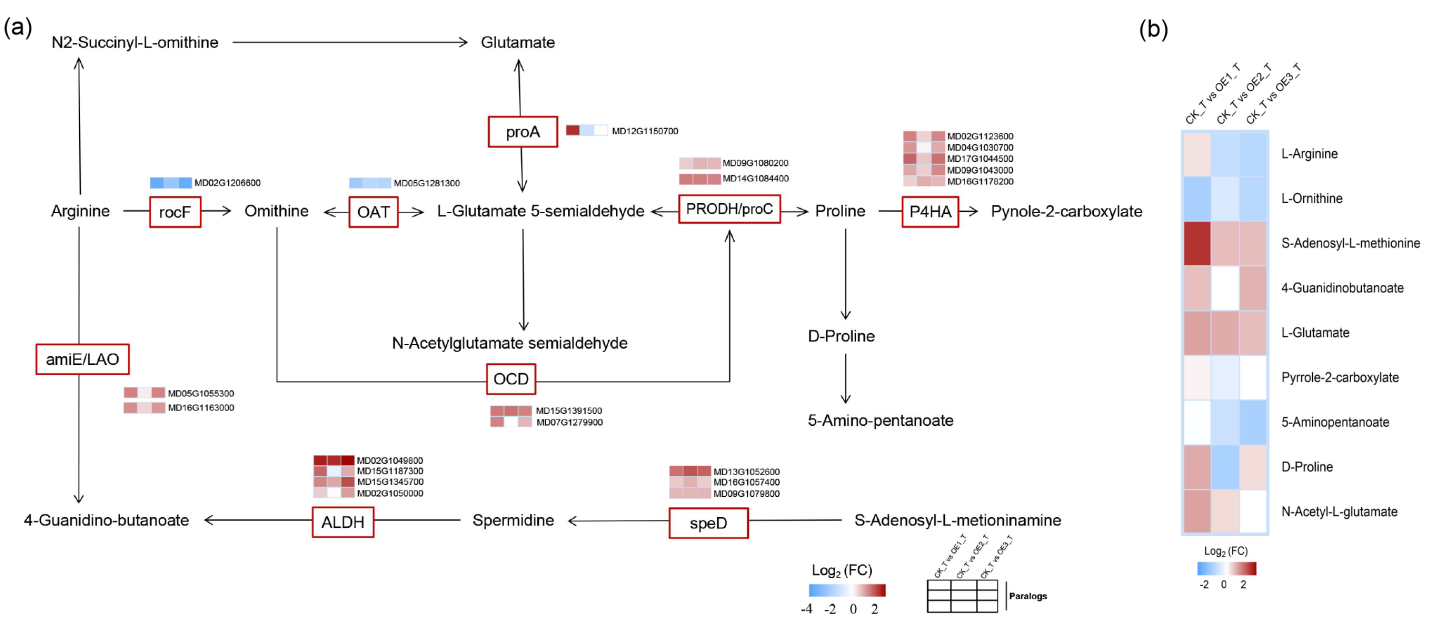
**

**Fig. S6** MdNAC104 is involved in the regulation of proline biosynthesis. (a) Relative expression levels of genes involved in the proline synthesis pathway. (b) Comparative analysis of the contents of proline synthesis-related metabolites in GL-3 and *MdNAC104* transgenic plants.

**
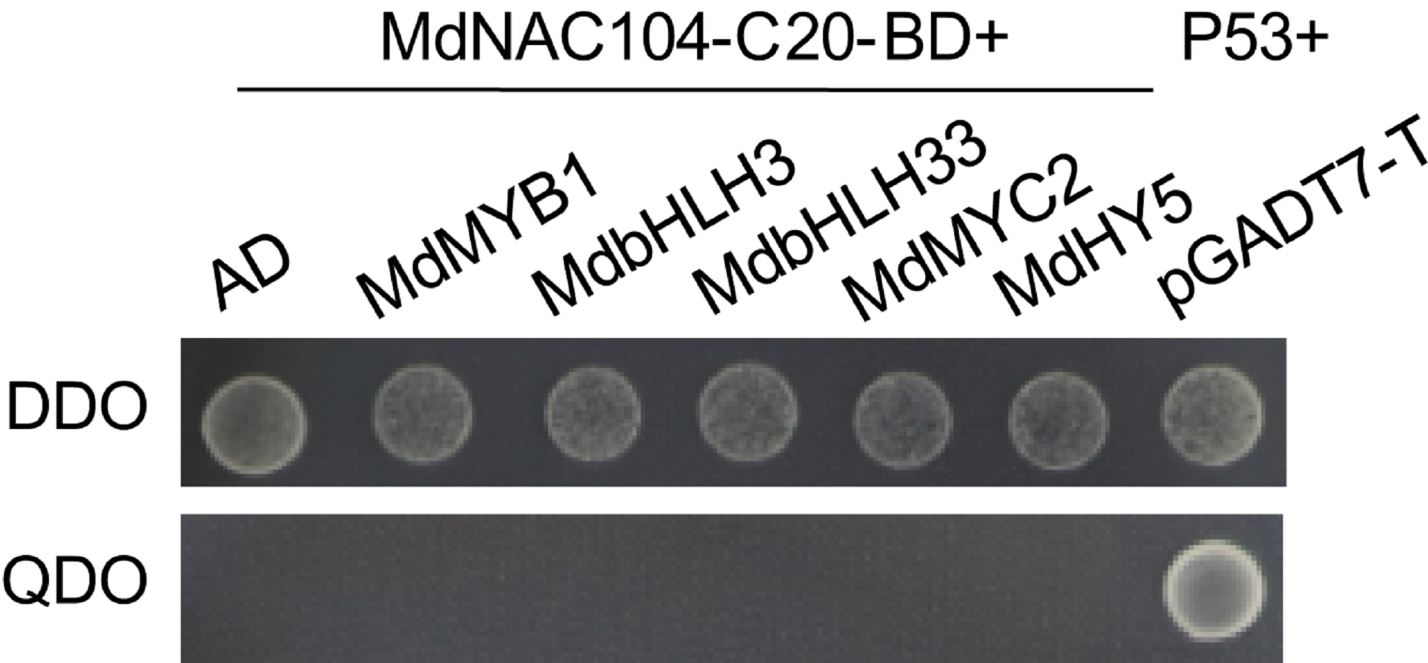
**

**Fig. S7** Protein interaction analysis between MdNAC104 and the transcription factors that promote anthocyanin accumulation in response to cold using the Y2H assay.

**
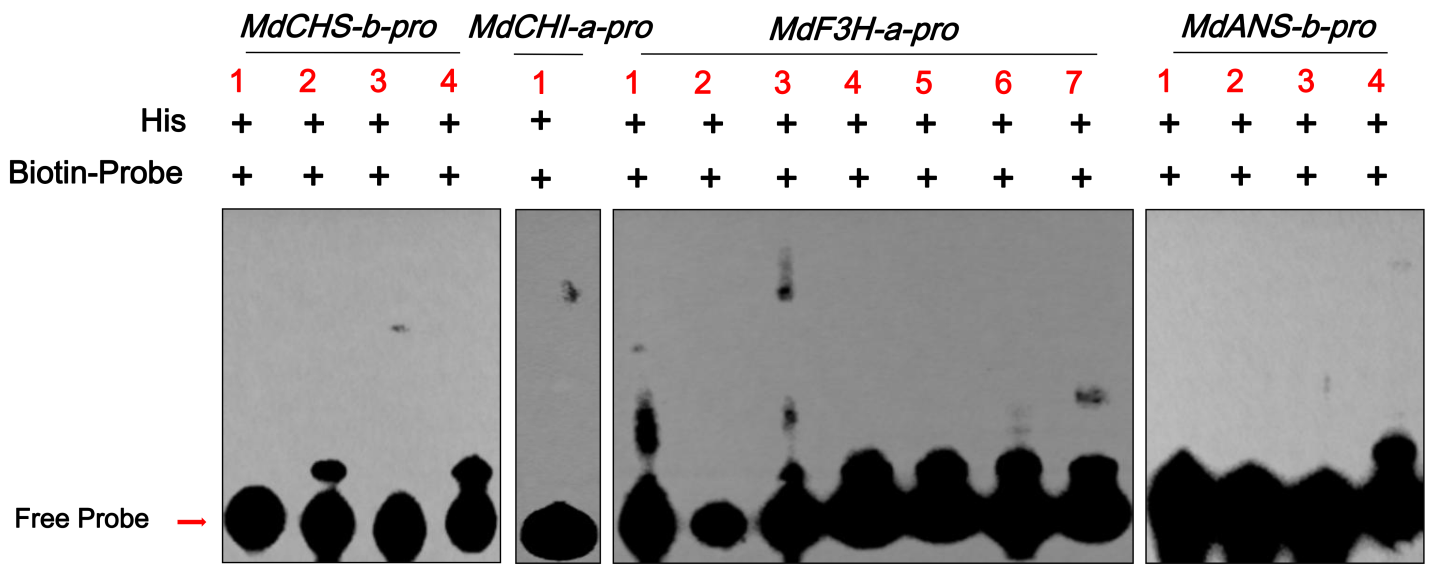
**

**Fig. S8** EMSA demonstrates binding specificity between MdNAC104 and the biotin-labeled probes. The His protein was used as the negative control, which was compared with the results in Fig. 6.
